# Using machine learning to predict student retention from socio-demographic characteristics and app-based engagement metrics

**DOI:** 10.1038/s41598-023-32484-w

**Published:** 2023-04-07

**Authors:** Sandra C. Matz, Christina S. Bukow, Heinrich Peters, Christine Deacons, Alice Dinu, Clemens Stachl

**Affiliations:** 1 Columbia University, New York, USA; 2 Ludwig Maximilian University of Munich, Munich, Germany; 3 Ready Education, Montreal, Canada; 4 University of St. Gallen, St. Gallen, Switzerland; 5 Montreal, Canada

**Keywords:** Psychology, Human behaviour

## Abstract

Student attrition poses a major challenge to academic institutions, funding bodies and students. With the rise of Big Data and predictive analytics, a growing body of work in higher education research has demonstrated the feasibility of predicting student dropout from readily available macro-level (e.g., socio-demographics or early performance metrics) and micro-level data (e.g., logins to learning management systems). Yet, the existing work has largely overlooked a critical meso-level element of student success known to drive retention: students’ experience at university and their social embeddedness within their cohort. In partnership with a mobile application that facilitates communication between students and universities, we collected both (1) institutional macro-level data and (2) behavioral micro and meso-level engagement data (e.g., the quantity and quality of interactions with university services and events as well as with other students) to predict dropout after the first semester. Analyzing the records of 50,095 students from four US universities and community colleges, we demonstrate that the combined macro and meso-level data can predict dropout with high levels of predictive performance (average AUC across linear and non-linear models = 78%; max AUC = 88%). Behavioral engagement variables representing students’ experience at university (e.g., network centrality, app engagement, event ratings) were found to add incremental predictive power beyond institutional variables (e.g., GPA or ethnicity). Finally, we highlight the generalizability of our results by showing that models trained on one university can predict retention at another university with reasonably high levels of predictive performance.

## Introduction

In the US, only about 60% of full-time students graduate from their program^1,2^ with the majority of those who discontinue their studies dropping out during their first year^3^ These high attrition rates pose major challenges to students, universities, and funding bodies alike^4,5^.

Dropping out of university without a degree negatively impacts students’ finances and mental health. Over 65% of US undergraduate students receive student loans to help pay for college, causing them to incur heavy debts over the course of their studies^6^. According to the U.S. Department of Education, students who take out a loan but never graduate are three times more likely to default on loan repayment than students who graduate^7^. This is hardly surprising, given that students who drop out of university without a degree, earn 66% less than university graduates with a bachelor's degree and are far more likely to be unemployed^2^. In addition to financial losses, the feeling of failure often adversely impacts students’ well-being and mental health^8^.

At the same time, student attrition negatively impacts universities and federal funding bodies. For universities, student attrition results in an average annual revenue reduction of approximately $16.5 billion per year through the loss of tuition fees^9,10^. Similarly, student attrition wastes valuable resources provided by states and federal governments. For example, the US Department of Education Integrated Postsecondary Education Data System (IPEDS) shows that between 2003 and 2008, state and federal governments together provided more than $9 billion in grants and subsidies to students who did not return to the institution where they were enrolled for a second year^11^.

Given the high costs of attrition, the ability to predict at-risk students – and to provide them with additional support – is critical^12,13^. As most dropouts occur during the first year^14^, such predictions are most valuable if they can identify at-risk students as early as possible^13,15,16^. The earlier one can identify students who might struggle, the better the chances that interventions aimed at protecting them from gradually falling behind – and eventually discontinuing their studies – will be effective^17,18^.

## Indicators of student retention

Previous research has identified various predictors of student retention, including previous academic performance, demographic and socio-economic factors, and the social embeddedness of a student in their home institution^19–23^.

Prior academic performance (e.g., high school GPA, SAT and ACT scores or college GPA) has been identified as one of the most consistent predictors of student retention: Students who are more successful academically are less likely to drop out^17,21,24–29^. Similarly, research has highlighted the role of demographic and socio-economic variables, including age, gender, and ethnicity^12,19,25,27,30^ as well as socio-economic status^31^ in predicting a students’ likelihood of persisting. For example, women are more likely to continue their studies than men^12,30,32,33^ while White and Asian students are more likely to persist than students from other ethnic groups^19,27,30^. Moreover, a student’s socio-economic status and immediate financial situation have been shown to impact retention. Students are more likely to discontinue their studies if they are first generation students^34–36^ or experience high levels of financial distress, (e.g., due to student loans or working nearly full time to cover college expenses)^37,38^. In contrast, students who receive financial support that does not have to be repaid post-graduation are more likely to complete their degree^39,40^.

While most of the outlined predictors of student retention are relatively stable intrapersonal characteristics and oftentimes difficult or costly to change, research also points to a more malleable pillar of retention: the students’ experience at university. In particular, the extent to which they are successfully integrated and socialized into the institution^16,22,41,42^. As Bean (2005) notes, “few would deny that the social lives of students in college and their exchanges with others inside and outside the institution are important in retention decisions” (p. 227)^41^. The extent to which a student is socially integrated and embedded into their institution has been studied in a number of ways, relating retention to the development of friendships with fellow students^43^, the student’s position in the social networks^16,29^, the experience of social connectedness^44^ and a sense of belonging^42,45,46^. Taken together, these studies suggest that interactions with peers as well as faculty and staff – for example through participation in campus activities, membership of organizations, and the pursuit of extracurricular activities – help students better integrate into university life^44,47^. In contrast, a lack of social integration resulting from commuting (i.e., not living on campus with other students) has shown to negatively impact a student’s chances of completing their degree^48–51^. In short, the stronger a student is embedded and feels integrated into the university community – particularly in their first year – the less likely the student will drop out^42,52^.

### Predicting retention using machine learning

A large proportion of research on student attrition has focused on understanding and explaining drivers of student retention. However, alongside the rise of computational methods and predictive modeling in the social sciences^53–55^, educational researchers and practitioners have started exploring the feasibility and value of data-driven approaches in supporting institutional decision making and educational effectiveness (for excellent overviews of the growing field see^56,57^). In line with this broader trend, a growing body of work has shown the potential of predicting student dropout with the help of machine learning. In contrast to traditional inferential approaches, machine learning approaches are predominantly concerned with predictive performance (i.e., the ability to accurately forecast behavior that has not yet occurred)^54^. In the context of student retention this means: How accurately can we predict whether a student is going to complete or discontinue their studies (in the future) by analyzing their demographic and socio-economic characteristics, their past and current academic performance, as well as their current embeddedness in the university system and culture?

Echoing the National Academy of Education’s statement (2017) that “in the educational context, big data typically take the form of administrative data and learning process data, with each offering their own promise for educational research” (p.4)^58^, the vast majority of existing studies have focused on the prediction of student retention from demographic and socio-economic characteristics as well as students’ academic history and current performance^13,59–66^. In a recent study, Aulck and colleagues trained a model on the administrative data of over 66,000 first-year students enrolled in a public US university (e.g., race, gender, highschool GPA, entrance exam scores and early college performance/transcript data) to predict whether they would re-enroll in the second year and eventually graduate^59^. Specifically, they used a range of linear and non-linear machine learning models (e.g., regularized logistic regression, k-nearest neighbor, random forest, support vector machine, and gradient boosted trees) to predict retention out-of-sample using a standard cross-validation procedures. Their model was able to predict dropouts with an accuracy of 88% and graduation with an accuracy of 81% (where 50% is chance).

While the existing body of work provides robust evidence for the potential of predictive models for identifying at-risk students, it is based on similar sets of macro-level data (e.g., institutional data, academic performance) or micro-level data (e.g., click-stream data). Almost entirely absent from this research is data on students’ daily experience and engagement with both other students and the university itself (meso-level). Although there have been a small number of studies trying to capture part of this experience by inferring social networks from smart card transactions that were made by students in the same time and place^16^ or engagement metrics with an open online course^67^, none of the existing work has offered a more holistic and comprehensive view on students’ daily experience. One potential explanation for this gap is that information about students’ social interactions with classmates or their day-to-day engagement with university services and events is difficult to track. While universities often have access to demographic or socio-economic variables through their Student Information Systems (SISs), and can easily track their academic performance, most universities do not have an easy way of capturing student’s deeper engagement with the system.

## Research overview

In this research, we partner with an educational software company – READY Education – that offers a virtual one-stop interaction platform in the form of a smartphone application to facilitate communication between students, faculty, and staff. Students receive relevant information and announcements, can manage their university activities, and interact with fellow students in various ways. For example, the app offers a social media experience like Facebook, including private messaging, groups, public walls, and friendships. In addition, it captures students’ engagement with the university asking them to check into events (e.g., orientation, campus events, and student services) using QR code functionality and prompting them to rate their experience afterwards (see Methods for more details on the features we extracted from this data). As a result, the READY Education app allows us to observe a comprehensive set of information about students that include both (i) institutional data (i.e., demographic, and socio-economic characteristics as well as academic performance), and (ii) their idiosyncratic experience at university captured by their daily interactions with other students and the university services/events. Combining the two data sources captures a student’s profile more holistically and makes it possible to consider potential interactions between the variable sets. For example, being tightly embedded in a social support network of friends might be more important for retention among first-generation students who might not receive the same level of academic support or learn about implicit academic norms and rules from their parents.

Building on this unique dataset, we use machine learning models to predict student retention (i.e., dropout) from both institutional and behavioral engagement data. Given the desire to identify at-risk students as early as possible, we only use information gathered in the students’ first semester to predict whether the student dropped out at any point in time during their program. To thoroughly validate and scrutinize our analytical approach, generate insights for potential interventions, and probe the generalizability of our predictive models across different universities, we investigate the following three research questions:

1. How accurately can we predict a student's likelihood of discontinuing their studies using information from the first term of their studies (i.e., institutional data, behavioral engagement data, and a combination of both)?
2. Which features are the most predictive of student retention?
3. How well do the predictive models generalize across universities (i.e., how well can we predict student retention of students from one university if we use the model trained on data from another university and vice versa)?

## Method

### Participants

We analyze de-identified data from four institutions with a total of 50,095 students (min = 476, max = 45,062). All students provided informed consent to the use of the anonymized data by READY Education and research partners. All experimental protocols were approved by the Columbia University Ethics Board, and all methods carried out were in accordance with the Board’s guidelines and regulations. The data stem from two sources: (a) Institutional data and (b) behavioral engagement data. The institutional data collected by the universities contain socio-demographics (e.g., gender, ethnicity), general study information (e.g., term of admission, study program), financial information (e.g., pell eligibility), students’ academic achievement scores (e.g., GPA, ACT) as well as the retention status. The latter indicated whether students continued or dropped out and serves as the outcome variable. As different universities collect different information about their students, the scope of institutional data varied between universities. Table 1 provides a descriptive overview of the most important sociodemographic characteristics for each of the four universities. In addition, it provides a descriptive overview of the app usage, including the average number of logs per student, the total number of sessions and logs, as well as the percentage of students in a cohort using the app (i.e., coverage). The broad coverage of students using the app, ranging between 70 and 98%, results in a largely representative sample of the student populations at the respective universities.

**Table 1:** Descriptive statistics of socio-demographic characteristics across the four universities.

	University 1	University 3	University 3	University 4
N students	476	2,010	2,547	45,062
% Drop out	25%	16%	16%	10.2%
% female	65%	61%	70%	65%
Ethnicity				
American Indian/Alaskan Native	1%	< 1%	< 1%	< 1%
Asian	1%	2%	3%	2%
Black/African American	8%	4%	27%	10%
Hispanic	9%	< 1%	6%	6%
Native Hawaiian/Pacific Islander	0%	< 1%	0%	< 1%
Nonresident Alien	3%	2%	< 1%	< 1%
Multiracial	5%	4%	3%	4%
Unknown	0%	4%	11%	1%
White	74%	85%	50%	77%
GPA	2.89 (0.98)	3.13 (0.78)	3.02 (0.88)	2.74 (1.25)
Pell-eligible	–	26%	80%	58%
First-generation	–	–	36%	–
Living in Residence	97%	90%	–	–
Data window	2018–2019	2016–2019	2016–2020	2019–2020
# Logs/student	1360 (1380)	626 (713)	197 (520)	328 (555)
Total sessions	162,462	296,546	155,386	6,863,236
Total logs	648,735	1,276,146	418,336	14,689,416
Coverage (% of overall cohort)	98%	97%	70%	87%

Notably, Universities 1–3 are traditional university campuses, while University 4 is a combination of 16 different community colleges. Given that there is considerable heterogeneity across campuses, the predictive accuracies for University 4 are a-priori expected to be lower than those observed for universities 1–3 (and partly speak to the generalizability of findings already). The decision to include University 4 as a single entity was based on the fact that separating out the 16 colleges would have resulted in an over-representation of community colleges that all share similar characteristics thereby artificially inflating the observed cross-university accuracies. Given these limitations (and the fact that the University itself collapsed the college campuses for many of their internal reports), we decided to analyze it as a single unit, acknowledging that this approach brings its own limitations.

The behavioral engagement data were generated through the app (see Table 1 for the specific data collection windows at each University). Behavioral engagement data were available in the form of time-stamped event-logs (i.e., each row in the raw data represented a registered event such as a tab clicked, a comment posted, a message sent). Each log could be assigned to a particular student via an anonymized, unique identifier. Across all four universities, the engagement data contained 7,477,630 sessions (Mean = 1,869,408, SD = 3,329,852) and 17,032,633 logs (Mean = 4,258,158, SD = 6,963,613) across all universities. For complete overview of all behavioral engagement metrics including a description see Table S1 in the Supplementary Materials.

### Analysis

#### Pre-processing and feature extraction

As a first step, we cleaned both the institutional and app data. For the institutional data, we excluded students who did not use the app and therefore could not be assigned a unique identifier. In addition, we excluded students without a term of admission to guarantee that we are only observing the students’ first semester. Lastly, we removed duplicate entries resulting from dual enrollment in different programs. For the app usage data, we visually inspected the variables in our data set for outliers that might stem from technical issues. We pre-processed data that reflected clicking through the app, named “clicked_[…]” and “viewed_[…]” (see Table S1 in the Supplementary Materials). A small number of observations showed unrealistically high numbers of clicks on the same tab in a very short period, which is likely a reflection of a student repeatedly clicking on a tab due to long loading time or other technical issues. To avoid oversampling these behaviors, we removed all clicks of the same type which were made by the same person less than one minute apart.

We extracted up to 462 features for each university across two broad categories: (i) institutional features and (ii) engagement features, using evidence from previous research as a reference point (see Table S2 in the Supplementary Materials for a comprehensive overview of all features and their availability for each of the universities). Institutional features contain students’ demographic, socio-economic and academic information. The engagement features represent the students’ behavior during their first term of studies. They can be further divided into app engagement and community engagement. The app engagement features represent the students’ behavior related to app usage, such as whether the students used the app before the start of the semester, how often they clicked on notifications or the community tabs, or whether their app use increased over the course of the semester. The community involvement features reflect social behavior and interaction with others, e.g., the number of messages sent, posts and comments made, events visited or a student’s position in the network as inferred from friendships and direct messages. Importantly, many of the features in our dataset will be inter-correlated. For example, living in college accommodation could signal higher levels of socio-economic status, but also make it more likely that students attend campus events and connect with other students living on campus. While intercorrelations among predictors is a challenge with standard inferential statistical techniques such as regression analyses, the methods we apply in this paper can account for a large number of correlated predictors.

Institutional features were directly derived from the data recorded by the institutions. As noted above, not all features were available for all universities, resulting in slightly different feature sets across universities. The engagement features were extracted from the app usage data. As we focused on an early prediction of drop-out, we restricted the data to event-logs that were recorded in the respective students' first term. Notably, the data captures students’ engagement as a time-stamped series of events, offering fine-grained insights into their daily experience. For reasons of simplicity and interpretability (see research question 2), we collapse the data into a single entry for each student. Specifically, we describe a student’s overall experience during the first semester, by calculating distribution measures for each student such as the arithmetic mean, standard deviation, kurtosis, skewness, and sum values. For example, we calculate how many daily messages a particular student sent or received during their first semester, or how many campus events they attended in total. However, we also account for changes in a student’s behavior over time by calculating more complex features such as entropy (e.g., the extent to which a person has frequent contact with few people or the same degree of contact with many people) and the development of specific behaviors over time measured by the slope of regression analyses, as well as features representing the regularity of behavior (e.g., the deviation of time between sending messages). Overall, the feature set was aimed at describing a student’s overall engagement with campus resources and other students during the first semester as well as changed in engagement over time. Finally, we extracted some of the features separately for weekdays and weekends to account for differences and similarities in students’ activities during the week and the weekend. For example, little social interaction on weekdays might predict retention differently than little social interaction on the weekend.

We further cleaned the data by discarding participants for whom the retention status was missing and those in which 95% or more of the values were zero or missing. Furthermore, features were removed if they showed little or no variance across participants, which makes them essentially meaningless in a prediction task. Specifically, we excluded numerical features which showed the same values for more than 90% of observations and categorical features which showed the same value for all observations.

In addition to these general pre-processing procedures, we integrated additional pre-processing steps into the resampling prior to training the models to avoid an overestimation of model performance^68^. To prevent problems with categorical features that occur when there are fewer levels in the test than in the training data, we first removed categories that did not occur in the training data. Second, we removed constant categorical features containing a single value only (and therefore no variation). Third, we imputed missing values using the following procedures: Categorical features were imputed with the mode. Following commonly used approaches to dealing with missing data, the imputation of numeric features varied between the learners. For the elastic net, we imputed those features with the median. For the random forest, we used twice the maximum to give missing values a distinct meaning that would allow the model to leverage this information. Lastly, we used the "Synthetic Minority Oversampling Technique" (SMOTE) to create artificial examples for the minority class in the training data^69^. The only exception was University 4 which followed a different procedure due to the large sample size and estimated computing power for implementing SMOTE. Instead of oversampling minority cases, we downsampled majority cases such that the positive and negative class were balanced. This was done to address the class imbalance caused by most students continuing their studies rather than dropping out^12^.

#### Predictive modeling approach

We predicted the retention status (1 = dropped out, 0 = continued) in a binary prediction task, with three sets of features: (1) institutional features (2) engagement features, and (3) a combined set of all features. To ensure the robustness of our predictions and to identify the model which is best suited for the current prediction context^54^, we compared a linear classifier (*elastic net;* implemented in glmnet 4.1–4)^70,71^ and a nonlinear classifier (*random forest*; implemented in randomForest 4.7–1)^72,73^. Both models are particularly well suited for our prediction context and are common choices in computational social science. That is, simple linear or logistic regression models are not suitable to work with datasets that have many inter-correlated predictors (in our case, a total of 462 predictors many of which are highly correlated) due to a high risk of overfitting. Both the elastic net and the random forest algorithm can effectively utilize large feature sets while reducing the risk of overfitting. We evaluate the performance of our six models for each school (2 algorithms and 3 feature sets), using out-of-sample benchmark experiments that estimate predictive performance and compare it against a common non-informative baseline model. The baseline represents a null-model that does not include any features, but instead always predicts the majority class, which in our samples means “continued.”^74^ Below, we provide more details about the specific algorithms (i.e., elastic net and random forest), the cross-validation procedure, and the performance metrics we used for model evaluation.

#### Elastic net model

The elastic net is a regularized regression approach that combines advantages of ridge regression^75^ with those of the LASSO^76^ and is motivated by the need to handle large feature sets. The elastic net shrinks the beta-coefficients of features that add little predictive value (e.g., intercorrelated, little variance). Additionally, the elastic net can effectively remove variables from the model by reducing the respective beta coefficients to zero^70^. Unlike classical regression models, the elastic net does not aim to optimize the sum of least squares, but includes two penalty terms (L1, L2) that incentivize the model to reduce the estimated beta value of features that do not add information to the model. Combining the L1 (the sum of absolute values of the coefficients) and L2 (the sum of the squared values of the coefficients) penalties, elastic net addresses the limitations of alternative linear models such as LASSO regression (not capable of handling multi-collinearity) and Ridge Regression (may not produce sparse-enough solutions)^70^.

Formally, following Hastie & Qian (2016) the model equation of elastic net for binary classification problems can be written as follows^77^. Suppose the response variable takes values in G = {0,1}, y_i_ denoted as I(g_i_ = 1), the model formula is written as

$$Pr(G=1|X=x)=\frac{{e}^{{\beta }_{0}+{\beta }^{T}x}}{1+{e}^{{\beta }_{0}+{\beta }^{T}x}},$$

After applying the log-odds transformation, the model formula can be written as

$$\frac{\mathrm{log}\left({\text{Pr}}\left(G=1|X=x\right)\right)}{{\text{Pr}}\left(G=0|X=x\right)}={\beta }_{0}+{\beta }^{T}x,$$

The objective function for logistic regression is the penalized negative binomial log-likelihood

$$\underset{({\beta }_{0},\beta )\in {\mathbb{R}}^{p+1}}{min}-[\frac{1}{N}\sum_{i=1}^{N}{y}_{i}\cdot ({\beta }_{0}+{x}_{i}^{T}\beta )-\mathrm{log}(1+{e}^{({\beta }_{0}+{x}_{i}^{T}\beta )})]+\lambda [(1-\alpha )\| \beta {\| }_{2}^{2}/2+\alpha \| \beta {\| }_{1}].$$

where λ is the regularization parameter that controls the overall strength of the regularization, α is the mixing parameter that controls the balance between L1 and L2 regularization with α values closer to zero to result in sparser models (lasso regression α = 1, ridge regression α = 0). β represents coefficients of the regression model, ||β||_1_ is the is the L1 norm of the coefficients (the sum of absolute values of the coefficients), ||β||_2_ is the L2 norm of the coefficients (the sum of the squared values of the coefficients).

The regularized regression approach is especially relevant for our model because many of the app-based engagement features are highly correlated (e.g., the number of clicks is related to the number of activities registered in the app). In addition, we favored the elastic net algorithm over more complex alternatives, because the regularized beta coefficients can be interpreted as feature importance, allowing insights into which predictors are most informative of college dropout^78,79^.

#### Random forest model

Random forest models are a widely used ensemble learning method that grows many bagged and decorrelated decision trees to come up with a “collective” prediction of the outcome (i.e., the outcome that is chosen by most trees in a classification problem)^72^. Individual decision trees recursively split the feature space (rules to distinguish classes) with the goal to separate the different classes of the criterion (drop out vs. remain in our case). For a detailed description of how individual decision trees operate and translate to a random forest see Pargent, Schoedel & Stachl^80^.

Unlike the elastic net, random forest models can account for nonlinear associations between features and criterion and automatically include multi-dimensional interactions between features. Each decision tree in a random forest considers a random subset of bootstrapped cases and features, thereby increasing the variance of predictions across trees and the robustness of the overall prediction. For the splitting in each node of each tree, a random subset of features (mtry hyperparameter that we optimize in our models) are used by randomly drawing from the total set. For each split, all combinations of split variables and split points are compared, with the model choosing the splits that optimize the separation between classes^72^.

The random forest algorithm can be formally described as follows (verbatim from Hastie et al., 2016, p. 588):

1. For b = 1 to B:
  1.1 Draw a bootstrap sample of size N from the training data.
  1.2 Grow a decision tree to the bootstrapped data, by recursively repeating the following steps for each terminal node of the tree, until the minimum node size is reached.
    1.2.1 Select m variables at random from the p variables.
    1.2.2 Pick the best variable/split-point among the m according to the loss function (in our case Gini-impurity decrease)
    1.2.3 Split the node into two daughter nodes.
2. Output the ensemble of trees
3. New predictions can then be made by generating a prediction for each tree and aggregating the results using majority vote.

The aggregation of predictions across trees in random forests improves the prediction performance compared to individual decision trees, as it can benefit from the trees’ variance and greatly reduces it to arrive at a single prediction^72,81^.

#### (Nested) Cross-validation: Out-of-sample model evaluation

**Figure 1:**
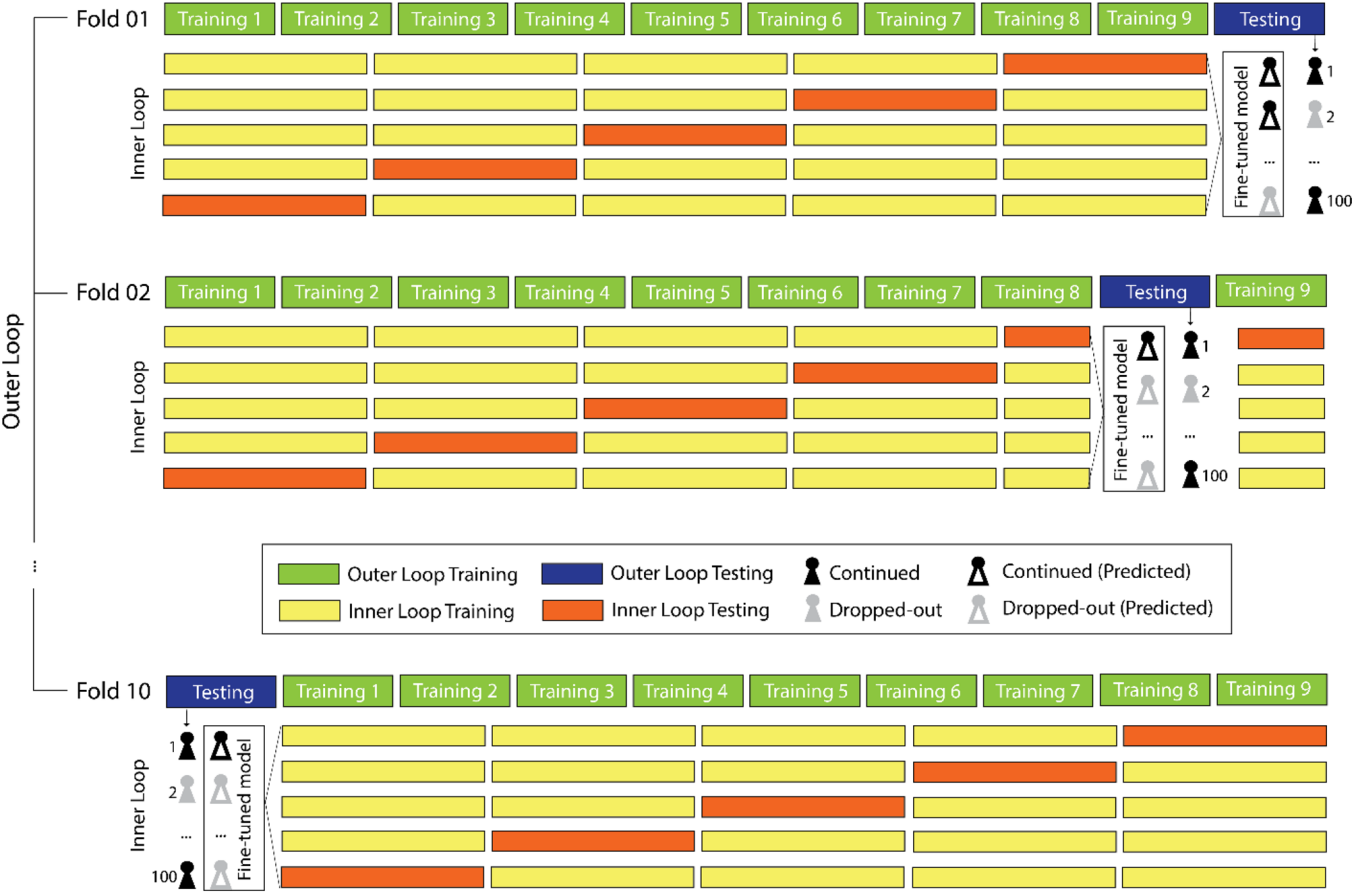
Schematic cross-validation procedure for out-of-sample predictions. The figure shows a tenfold cross-validation in the outer loop which is used to estimate the overall performance of the model by comparing the predicted outcomes for each student in the previously unseen test set with their actual outcomes. Within each of the 10 outer loops, a fivefold cross-validation in the inner loop is used to finetune model hyperparameters by evaluating different model settings.

We evaluate the performance of our predictive models using an out-of-sample validation approach. The idea behind out-of-sample validation is to increase the likelihood that a model will accurately predict student dropout on new data (e.g. new students) by using different datasets when training and evaluating the model. A commonly used, efficient technique for out-of-sample validation is to repeatedly fit (cf. training) and evaluate (cf. testing) models on non-overlapping parts of the same datasets and to combine the individual estimates across multiple iterations. This procedure – known as cross-validation – can also be used for model optimization (e.g., hyperparameter-tuning, pre-processing, variable selection), by repeatedly evaluating different settings for optimal predictive performance. When both approaches are combined, evaluation and optimization steps need to be performed in a nested fashion to ensure a strict separation of training and test data for a realistic out-of-sample performance estimation. The general idea is to emulate all modeling steps in each fold of the resampling as if it were a single in-sample model. Here, we use nested cross-validation to estimate the predictive performance of our models, to optimize model hyperparameters, and to pre-process data. We illustrate the procedure in Fig. 1.

The cross-validation procedure works as follows: Say we have a dataset with 1,000 students. In a first step, the dataset is split into ten different subsamples, each containing data from 100 students. In the first round, nine of these subsamples are used for training (i.e., fitting the model to estimate parameters, green boxes). That means, the data from the first 900 students will be included in training the model to relate the different features to the retention outcome. Once training is completed, the model’s performance can be evaluated on the data of the remaining 100 students (i.e., test dataset, blue boxes). For each student, the actual outcome (retained or discontinued, grey and black figures) is compared to the predicted outcome (retained or discontinued, grey and black figures). This comparison allows for the calculation of various performance metrics (see “Performance metrics” section below for more details). In contrast to the application of traditional inferential statistics, the evaluation process in predictive models separates the data used to train a model from the data used to evaluate these associations. Hence any overfitting that occurs at the training stage (e.g., using researcher degrees of freedom or due to the model learning relationships that are unique to the training data), hurts the predictive performance in the testing stage. To further increase the robustness of findings and leverage the entire dataset, this process is repeated for all 10 subsamples, such that each subsample is used nine times for training and once for testing. Finally, the obtained estimates from those ten iterations are aggregated to arrive at a cross-validated estimate of model performance. This tenfold cross validation procedure is referred to as the “outer loop”.

In addition to the outer loop, our models also contain an “inner loop”. The inner loop consists of an additional cross-validation procedure that is used to identify ideal hyperparameter settings (see “Hyperparameter tuning” section below). That is, in each of the ten iterations of the outer loop, the training sample is further divided into a training and test set to identify the best parameter constellations before model evaluation in the outer loop. We used fivefold cross-validation in the inner loop. All analyses scripts for the pre-processing and modeling steps are available on OSF (https://osf.io/bhaqp/?view_only=629696d6b2854aa9834d5745425cdbbc).

#### Performance metrics

We evaluate model performance based on four different metrics. Our main metric for model performance is AUC (area under the received operating characteristics curve). AUC is commonly used to assess the performance of a model over a 50%-chance baseline, and can range anywhere between 0 and 1. The AUC metric captures the area under the receiver operating characteristic (ROC) curve, which plots the true positive rate (TPR or recall; i.e. the percentage of correctly classified dropouts among all students who actually dropped out), against the false positive rate (FPR; i.e. the percentage of students erroneously classified as dropouts among all the students who actually continued). When the AUC is 0.5, the model’s predictive performance is equal to chance or a coin flip. The closer to 1, the higher the model’s predictive performance in distinguishing between students who continued and those who dropped out.

In addition, we report the F1 score, which ranges between 0 and 1^82^. The F1 score is based on the model’s positive predictive value (or precision, i.e., the percentage of correctly classified dropouts among all students predicted to have dropped out) as well as the model's TPR. A high F1 score hence indicates that there are both few false positives and few false negatives.

Given the specific context, we also report the TPR and the true negative rates (TNR, i.e. the percentage of students predicted to continue among all students who actually continued). Depending on their objective, universities might place a stronger emphasis on optimizing the TPR to make sure no student who is at risk of dropping out gets overlooked or on optimizing the TNR to save resources and assure that students do not get overly burdened. Notably, in most cases, universities are likely to strive for a balance between the two, which is reflected in our main AUC measure. All reported performance metrics represent the mean predictive performance across the 10 cross-validation folds of the outer loop^54^.

#### Hyperparameter tuning

We used a randomized search with 50 iterations and fivefold cross-validation for hyperparameter tuning in the inner loop of our cross-validation. The randomized search algorithm fits models with hyperparameter configurations randomly selected from a previously defined hyperparameter space and then picks the model that shows the best generalized performance averaged over the five cross-validation folds. The best hyperparamter configuration is used for training in the outer resampling loop to evaluate model performance.

For the elastic net classifier, we tuned the regularization parameter lambda, the decision rule used to choose lambda, and the L1-ratio parameter. The search space for lambda encompassed the 100 glmnet default values^71^. The space of decision rules for lambda included lambda.min which chooses the value of lambda that results in the minimum mean cross-validation error, and lambda.1se which chooses the value of lambda that results in the most regularized model such that the cross-validation error remains within one standard error of the minimum. The search space for the L1-ratio parameter included the range of values between 0 (ridge) to 1 (lasso). For the random forest classifier, we tuned the number of features selected for each split within a decision tree (mtry) and the minimum node size (i.e., how many cases are required to be left in the resulting end-nodes of the tree). The search space for the number of input features per decision tree was set to a range of 1 to p, where p represents the dimensionality of the feature space. The search space for minimum node size was set to a range of 1 to 5. Additionally, for both models, we tuned the oversampling rate and the number or neighbors used to generate new samples utilized by the SMOTE algorithm. The oversampling rate was set to a range of 2 to 15 and the number of nearest neighbors was set to a range of 1 to 10.

## Results

### RQ1: How accurately can we predict a student's likelihood of discontinuing their studies using information from the first term of their studies?

**Figure 2:**
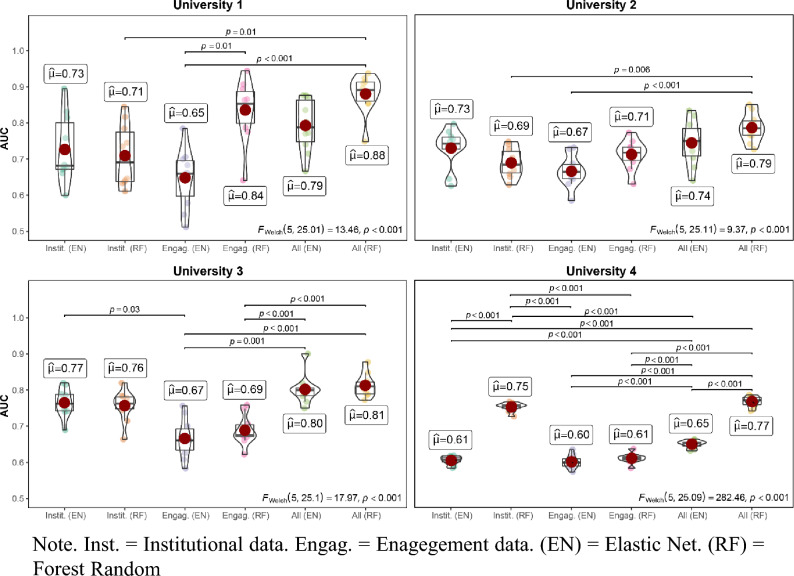
AUC performance across the four universities for different feature sets and model. Inst. = Institutional data. Engag. = Engagement data. (EN) = Elastic Net. (RF) = Random Forest.

Figure 2 displays AUC scores (Y-axis) across the different universities (rows), separated by the different feature sets (colors) and predictive algorithms (X-axis labels). The figure displays the distribution of AUC accuracies across the 10 cross-validation folds, alongside their mean and standard deviation. Independent t-tests using Holm corrections for multiple comparisons indicate statistical differences in the predictive performance across the different models and feature sets within each university. Table 2 provides the predictive performance across all four metrics.

**Table 2:** Indicators of predictive performance across different universities, models, feature sets and performance metrics.

University	Feature Set	Elastic net				Random forest			
		F1	AUC	TPR	TNR	F1	AUC	TPR	TNR
University 1	All	0.55	0.79	0.61	0.80	0.66	0.88	0.65	0.90
University 1	Engagement	0.44	0.65	0.71	0.51	0.61	0.84	0.59	0.90
University 1	Institutional	0.46	0.73	0.58	0.71	0.50	0.71	0.66	0.66
University 2	All	0.39	0.74	0.47	0.82	0.40	0.79	0.38	0.90
University 2	Engagement	0.34	0.67	0.68	0.57	0.32	0.71	0.40	0.79
University 2	Institutional	0.40	0.73	0.47	0.84	0.37	0.69	0.50	0.78
University 3	All	0.46	0.80	0.51	0.88	0.46	0.81	0.39	0.95
University 3	Engagement	0.34	0.67	0.69	0.56	0.33	0.69	0.59	0.63
University 3	Institutional	0.47	0.77	0.50	0.88	0.44	0.76	0.51	0.85
University 4	All	0.23	0.65	0.72	0.49	0.35	0.77	0.59	0.79
University 4	Engagement	0.21	0.60	0.67	0.47	0.22	0.61	0.60	0.56
University 4	Institutional	0.21	0.61	0.82	0.33	0.33	0.75	0.58	0.78

*F1*F1 score, *AUC* Area under ROC-curve, *TPR* True positive rate, *TNR* True negative rate. By setting the predicted outcome to the majority class, the featureless baseline learner produces F1 = 0, AUC = 0.5, TPR = 0 and TNR = 1 across all models.

Overall, our models showed high levels of predictive accuracies across universities, models, feature sets and performance metrics, significantly outperforming the baseline in all instances. The main performance metric AUC reached an average of 73% (where 50% is chance), with a maximum of 88% for the random forest model and the full feature set in University 1. Both institutional features and engagement features significantly contributed to predictive performance, highlighting the fact that a student’s likelihood to drop out is both a function of their more stable socio-demographic characteristics as well as their experience of campus life. In most cases, the joint model (i.e., the combination of institutional and engagement features) performed better than each of the individual models alone. Finally, the random forest models produced higher levels of predictive performance than the elastic net in most cases (average AUC elastic net = 70%, AUC random forest = 75%), suggesting that the features are likely to interact with one another in predicting student retention and might not always be linearly related to the outcome.

### RQ2: Which features are the most predictive of student retention?

To provide insights into the underlying relationships between student retention and socio-demographic as well as behavioral features, we examined two indicators of feature importance that both offer unique insights. First, we calculated the zero-order correlations between the features and the outcome for each of the four universities. We chose zero-order correlations over elastic net coefficients as they represent the relationships unaltered by the model’s regularization procedure (i.e., the relationship between a feature and the outcome is shown independently of the importance of the other features in the model). To improve the robustness of our findings, we only included the variables that passed the threshold for data inclusion in our models and had less than 50% of the data imputed. The top third of Table 3 displays the 10 most important features (i.e., highest absolute correlation with retention). The sign in brackets indicates the direction of the effects with ( +) indicating a protective factor and (−) indicating a risk factor. Features that showed up in the top 10 for more than 1 university are highlighted in bold.

**Table 3:**
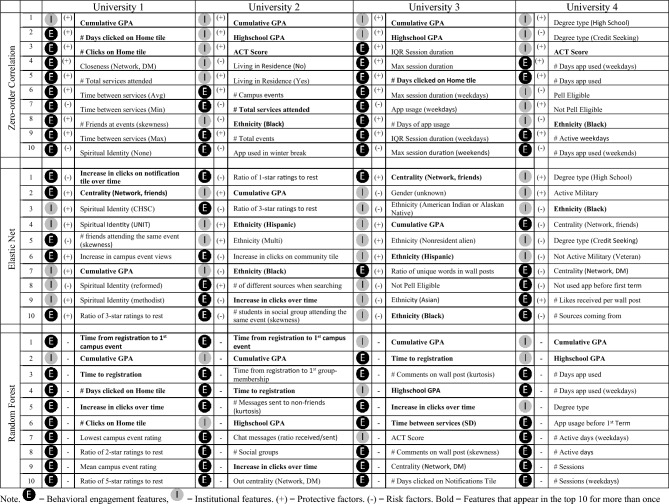
Overview of the ten most important features in the elastic net (top) and random forest (bottom) models.

Second, we calculated permutation variable importance scores for the elastic net and random forest models. For the elastic net model, feature importance is reported as the model coefficient after shrinking the coefficients according to their incremental predictive power. Compared to the zero-order correlation, the elastic net coefficients hence identify the features that have the strongest unique variance. For the random forest models, feature importance is reported as a model-agnostic metric that estimates the importance of a feature by observing the drop in model predictive performance when the actual association between the feature and the outcome is broken by randomly shuffling observations^72,83^. A feature is considered important if shuffling its values increases the model error (and therefore decreases the model’s predictive performance). In contrast to the coefficients from the elastic net model, the permutation feature importance scores are undirected and do not provide insights into the specific nature of the relationship between the feature and the outcome. However, they account for the fact that some features might not be predictive themselves but could still prove valuable in the overall model performance because they moderate the impact of other features. For example, minority or first-generation students might benefit more from being embedded in a strong social network than majority students who do not face the same barriers and are likely to have a stronger external support network. The bottom of Table 3 displays the 10 most important features in the elastic net and random forest models (i.e., highest permutation variable importance).

Supporting the findings reported in RQ1, the zero-order correlations confirm that both institutional and behavioral engagement features play an important role in predicting student retention. Aligned with prior work, students’ performance (measured by GPA or ACT) repeatedly appeared as one of the most important predictors across universities and models. In addition, many of the engagement features (e.g., services attended, chat messages network centrality) are related to social activities or network features, supporting the notion that a student’s social connections and support play a critical role in student retention. In addition, the extent to which students are positively engaged with their institutions (e.g., by attending events and rating them highly) appears to play a critical role in preventing dropout.

### RQ3: How well do the predictive models generalize across universities?

**Figure 3:**
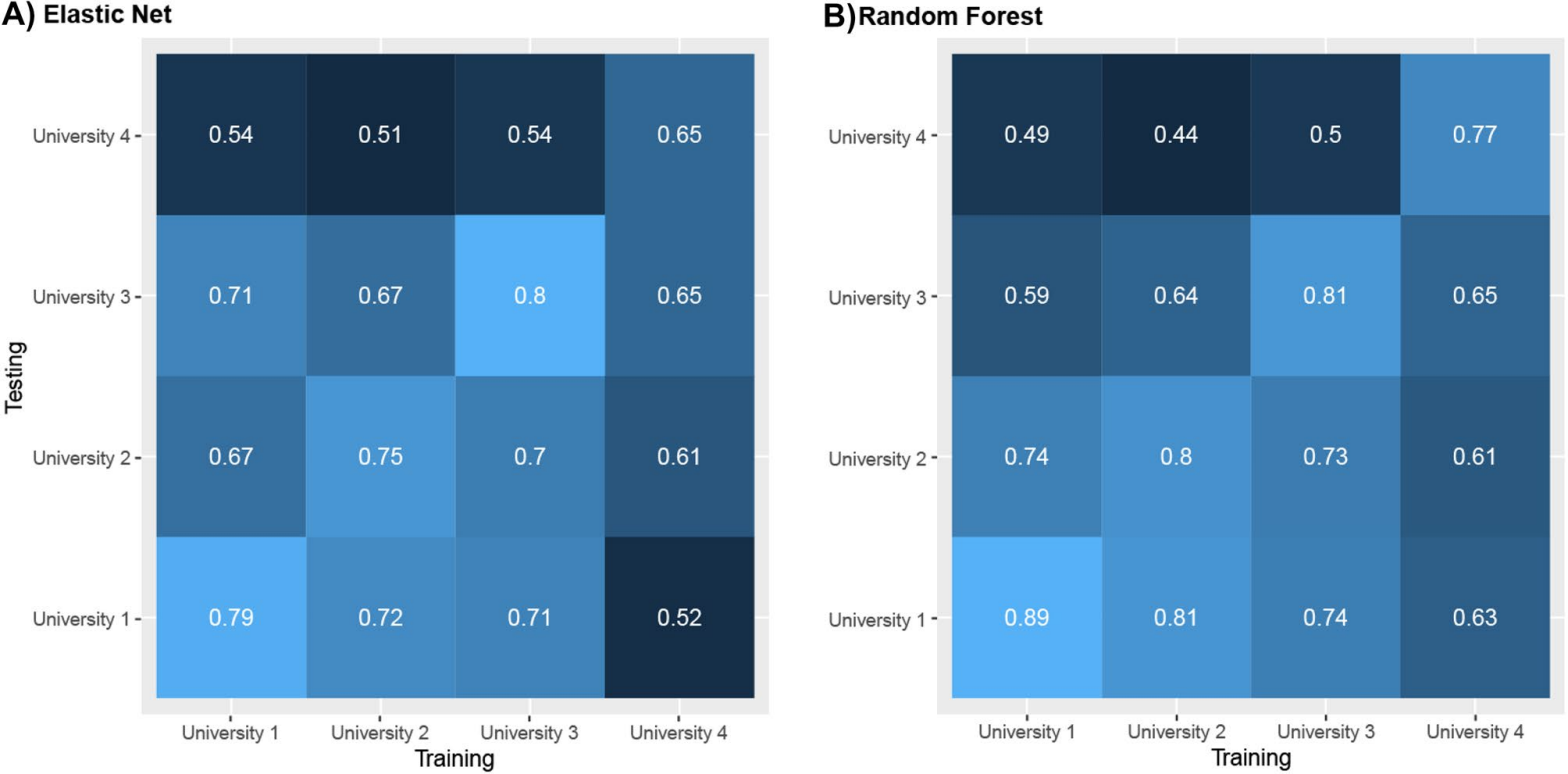
Performance (average AUC) of cross-university predictions.

To test the generalizability of our models across universities, we used the predictive model trained on one university (e.g., University 1) to predict retention of the remaining three universities (e.g., Universities 2–4). Figures 3A,B display the AUCs across all possible pairs, indicating which university was used for training (X-axis) and which was used for testing (Y-axis, see Figure S1 in the SI for graphs illustrating the findings for F1, TNR and TPR).

Overall, we observed reasonably high levels of predictive performance when applying a model trained on one university to the data of another. The average AUC observed was 63% (for both the elastic net and the random forest), with the highest predictive performance reaching 74% (trained on University 1, predicting University 2), just 1%-point short of the predictive performance observed for the prediction from the universities own model (trained on University 2, predicting University 2). Contrary to the findings in RQ1, the random forest models did not perform better than the elastic net when making predictions for other universities. This suggests that the benefits afforded by the random forest models capture complex interaction patterns that are somewhat unique to each university but might not generalize well to new contexts. The main outlier in generalizability was University 4, where none of the other models reached accuracies much better than chance, and whose model produced relatively low levels of accuracies when predicting student retention across universities 1–2. This is likely a result of the fact that University 4 was qualitatively different from the other universities in several ways, including the fact that University 4 was a community college and consisted of 16 different campuses that were merged for the purpose of this analysis (see Methods for more details).

## Discussion

We show that student retention can be predicted from institutional data, behavioral engagement data, and their combination. Using data from over 50,000 students across four Universities, our predictive models achieve out-of-sample accuracies of up to 88% (where 50% is chance). Notably, while both institutional data and behavioral engagement data significantly predict retention, the combination of the two performs best in most instances. This finding is further supported by our feature importance analyses which suggest that both institutional and behavioral engagement features are among the most important predictors of student retention. Specifically, academic performance as measured by GPA and behavioral metrics associated with campus engagement (e.g., event attendances or ratings) or a student’s position in the network (e.g., closeness or centrality) were shown to consistently act as protective factors. Finally, we highlight the generalizability of our models across universities. Models trained on one university were able to predict student retention at another with reasonably high levels of predictive performance. As one might expect, the generalizability across universities heavily depends on the extent to which the universities are similar on important structural dimensions, with prediction accuracies dropping radically in cases where similarity is low (see low cross-generalization for University 4).

### Contributions to the scientific literature

Our findings contribute to the existing literature in several ways. First, they respond to recent calls for more predictive research in psychology^54,55^ as well as the use of Big Data analytics in education research^56,57^. Not only do our models consider socio-demographic characteristics that are collected by universities, but they also capture students’ daily experience and university engagement by tracking behaviors via the READY Education app. Our findings suggest, these more psychological predictors of student retention can improve the performance of predictive models above and beyond socio-demographic variables. This is consistent with previous findings suggesting that the inclusion of engagement metrics improves the performance of predictive models^16,84,85^. Overall, our models showed superior accuracies to models of former studies that were trained only on demographics and transcript records^15,25^ or less comprehensive behavioral features^16^ and provided results comparable to those reported in studies that additionally included a wide range of socio-economic variables^12^. Given that the READY Education app captures only a fraction of the students' actual experience, the high predictive accuracies make an even stronger case for the importance of student engagement in college retention.

Second, our findings provide insights into the features that are most important in predicting whether a student is going to drop out or not. By doing so they complement our predictive approach with layers of understanding that are conducive to not only validating our models but also generating insights into potential protective and risk factors. Most importantly, our findings highlight the relevance of the behavioral engagement metrics for predicting student retention. Most features identified as being important in the prediction were related to app and community engagement. In line with previous research, features indicative of early and deep social integration, such as interactions with peers and faculty or the development of friendships and social networks, were found to be highly predictive^16,41^. For example, it is reasonable to assume that a short time between app registration and the first visit of a campus event (one of the features identified as important) has a positive impact on retention, because campus events offer ideal opportunities for students to socialize^86^. Early participation in a campus event implies early integration and networking with others, protecting students from perceived stress^87^ and providing better social and emotional support^88^. In contrast, a student who never attends an event or does so very late in the semester may be less connected to the campus life and the student community which in turn increases the likelihood of dropping out. This interpretation is strengthened by the fact that a high proportion of positive event ratings was identified as an important predictor of a student continuing their studies. Students who enjoy an event are likely to feel more comfortable, be embedded in the university life, make more connections, and build stronger connections. This might result in a virtuous cycle in which students continue attending events and over time create a strong social connection to their peers. As in most previous work, a high GPA score was consistently related to a higher likelihood of continuing one’s studies^21,24^. Although their importance varied across universities, ethnicity was also found to play a major role for retention, with consistent inequalities replicating in our predictive models^12,19,47^. For example, Black students were on average more likely to drop-out, suggesting that universities should dedicate additional resources to protect this group. Importantly, all qualitative interpretations are post-hoc. While many of the findings are intuitive and align with previous research on the topic, future studies should validate our results and investigate the causality underlying the effects in experimental or longitudinal within-person designs^54,78^.

Finally, our findings are the first to explore the extent to which the relationships between certain socio-demographic and behavioral characteristics might be idiosyncratic and unique to a specific university. By being able to compare the models across four different universities, we were able to show that many of the insights gained from one university can be leveraged to predict student retention at another. However, our findings also point to important boundary conditions: The more dissimilar universities are in their organizational structures and student experience, the more idiosyncratic the patterns between certain socio-demographic and behavioral features with student retention will be and the harder it is to merely translate general insights to the specific university campus.

### Practical contributions

Our findings also have important practical implications. In the US, student attrition results in an average annual revenue loss of approximately $16.5 billion per year^9,10^ and over $9 billion wasted in federal and state grants and subsidies that are awarded to students who do not finish their degree^11^. Hence, it is critical to predict potential dropouts as early and as accurately as possible to be able to offer dedicated support and allocate resources where they are needed the most. Our models rely exclusively on data collected in the first semester at university and are therefore an ideal “early warning” system for universities who want to predict whether their students will likely continue their studies or drop out at some point. Depending on the university’s resources and goals, the predictive models can be optimized for different performance measures. Indeed, a university might decide to focus on the true positive rate to capture as many dropouts as possible. While this would mean erroneously classifying “healthy “ students as potential dropouts, universities might decide that the burden of providing “unnecessary “ support to these healthy students is worth the reduced risk of missing a dropout. Importantly, our models go beyond mere socio-demographic variables and allow for a more nuanced, personal model that considers not just “who someone is” but also what their experience on campus looks like. As such, our models make it possible to acknowledge individuality rather than using over-generalized assessments of entire socio-demographic segments.

Importantly, however, it is critical to subject these models to continuous quality assurance. While predictive models could allow universities to flag at-risk students early, they could also perpetuate biases that get calcified in the predictive models themselves. For example, students who are traditionally less likely to discontinue their studies might have to pass a much higher level of dysfunctional engagement behavior before their file gets flagged as “at-risk”. Similarly, a person from a traditionally underrepresented group might receive an unnecessarily high volume of additional check-ins even though they are generally flourishing in their day-to-day experience. Given that being labeled as “at-risk” can be associated with stigma that could reinforce stigmas around historically marginalized groups, it will be critical to monitor both the performance of the model over time as well as the perception of its helpfulness among administrators, faculty, and students.

### Limitations and future research

Our study has several limitations and highlights avenues for future research. First, our sample consisted of four US universities. Thus, our results are not necessarily generalizable to countries with more collectivistic cultures and other education systems such as Asia, where the reasons for dropping out might be different^89,90^, or Europe where most students work part-time jobs and live off-campus. Future research should investigate the extent to which our models can generalize to other cultural contexts and identify the features of student retention that are universally valid across contexts.

Second, our predictive models relied on app usage data. Therefore, our predictive approach could only be applied to students who decided to use the app. This selection, in and by itself, is likely to introduce a sampling bias, as students who decide to use the app might be more likely to retain in the first place, restricting the variance in observations, and excluding students for whom app usage data was not available. However, as our findings suggest, the institutional data alone provide predictive performance independent of the app features, making this a viable alternative for students who do not use the app.

Third, our predictive models rely on cross-sectional predictions. That is, we observe a students’ behavior over the course of an entire semester and based on the patterns observed in other students we predict whether that student is likely to drop out or not. Future research could try to improve both the predictive performance of the model and its usefulness for applied contexts by modeling within-person trends dynamically. Given enough data, the model could observe a person’s baseline behavior and identify changes from that baseline as potentially problematic. In fact, more social contact with other students might be considered a protective factor in our cross-sectional model. However, there are substantial individual differences in how much social contact individuals seek out and enjoy^91^. Hence, sending 10 chat messages a week might be considered a lot for one person, but very little for another. Future research should hence investigate whether the behavioral engagement features allow for a more dynamic within-person model that makes it possible to take base rates into account and provide a dynamic, momentary assessment of a student’s likelihood to drop out.

Fourth, although the engagement data was captured as a longitudinal time series with time-stamped events, we collapsed the data into a single set of cross-sectional features for each student. Although some of these features captures variation in behaviors over time (e.g., entropy and linear trends), future research should try to implement more advanced machine learning models to account for this time series data directly. For example, long short-term memory models (LSTMs)^92^ – a type of recurrent neural network – are capable of learning patterns in longitudinal, sequential data like ours.

Fifth, even though the current research provides initial insights into the workings of the models by highlighting the importance of certain features, the conclusions that can be drawn from these analyses are limited as the importance metrics are calculated for the overall population. Future research could aim to calculate the importance of certain features at the individual level to test whether their importance varies across certain socio-demographic features. Estimating the importance of a person’s position in the social network on an individual level, for example, would make it possible to see whether the importance is correlated with institutional data such as minority or first-generation status.

Finally, our results lay the foundation for developing interventions that foster retention through shaping students’ experience at university^93^. Interventions which have been shown to have a positive effect on retention, include orientation programs and academic advising^94^, student support services like mentoring and coaching as well as need-based grants^95^. However, to date, the first-year experience programs meant to strengthen social integration of first year students, do not seem to have yielded positive results^96,97^. Our findings could support the development of interventions aimed at improving and maintaining student integration on campus. On a high level, the insights into the most important features provide an empirical path for developing relevant interventions that target the most important levers of student retention. For example, the fact that the time between registration and the first event attendance has such a big impact on student retention means that universities should do everything they can to get students to attend events as early as possible. Similarly, they could develop interventions that lead to more cohesive networks among cohorts and make sure that all students connect to their community. On a deeper, more sophisticated level, new approaches to model explainability could allow universities to tailor their intervention to each student^98,99^. For example, explainable AI makes it possible to derive decision rules for each student, indicating which features were critical in predicting the students’ outcome. While student A might be predicted to drop out because they are disconnected from the network, student B might be predicted to drop out because they don’t access the right information on the app. Given this information, universities would be able to personalize their offerings to the specific needs of the student. While student A might be encouraged to spend more time socializing with other students, student B might be reminded to check out important course information. Hence, predictive models could not only be used to identify students at risk but also provide an automated path to offering personalized guidance and support.

## Conclusion

For every study that is discontinued, an educational dream shatters. And every shattered dream has a negative long-term impact both on the student and the university the student attended. In this study we introduce an approach to accurately predicting student retention after the first term. Our results show that student retention can be predicted with relatively high levels of predictive performance when considering institutional data, behavioral engagement data, or a combination of the two. By combining socio-demographic characteristics with passively observed behavioral traces reflecting a student’s daily activities, our models offer a holistic picture of students' university experiences and its relation to retention. Overall, such predictive models have great potential both for the early identification of at-risk students and for enabling timely, evidence-based interventions.

## Supplementary Information

Supplementary Information.

## Data Availability

Raw data are not publicly available due to their proprietary nature and the risks associated with de-anonymization, but they are available from the corresponding author on reasonable request. The pre-processed data and all analyses codes are available on OSF (https://osf.io/bhaqp/) to facilitate reproducibility of our work. Data were analyzed using R, version 4.0.0 (R Core Team, 2020; see subsections for specific packages and versions used). The study’s design relies on secondary data and the analyses were not preregistered.
