# Author Correction: Using machine learning to predict student retention from socio-demographic characteristics and app-based engagement metrics

**DOI:** 10.1038/s41598-023-36579-2

**Published:** 2023-06-21

**Authors:** Sandra C. Matz, Christina S. Bukow, Heinrich Peters, Christine Deacons, Alice Dinu, Clemens Stachl

**Affiliations:** 1 Columbia University, New York, USA; 2 Ludwig Maximilian University of Munich, Munich, Germany; 3 Ready Education, Montreal, Canada; 4 Montreal, Canada; 5 University of St. Gallen, St. Gallen, Switzerland

Correction to: *Scientific Reports* 10.1038/s41598-023-32484-w, published online 07 April 2023

Alice Dinu was omitted from the author list in the original version of this Article.

The Author Contributions section now reads:

“S.C.M., C.B, A.D., H.P., and C.S. designed the research. C.D. and A.D. provided the data. S.C.M, C.B. and H.P. analyzed the data. S.C.M and C.B. wrote the manuscript. All authors reviewed the manuscript. Earlier versions of this research were part of the C.B.’s masters thesis which was supervised by S.C.M. and C.S.”

Additionally, in the Data availability section, the OSF repository link was incorrect.

“The pre-processed data and all analyses codes are available on OSF (https://osf.io/bhaqp/?view_only=629696d6b2854aa9834d5745425cdbbc) to facilitate reproducibility of our work. Data were analyzed using R, version 4.0.0 (R Core Team, 2020; see subsections for specific packages and versions used).”

now reads:

“The pre-processed data and all analyses codes are available on OSF (https://osf.io/bhaqp/) to facilitate reproducibility of our work. Data were analyzed using R, version 4.0.0 (R Core Team, 2020; see subsections for specific packages and versions used).”

Finally, the legend of Figure 2 was incomplete.

“AUC performance across the four universities for different feature sets and models.”

now reads:

“AUC performance across the four universities for different feature sets and model. Inst. = Institutional data. Engag. = Engagement data. (EN) = Elastic Net. (RF) = Random Forest"

The original Article and Supplementary Information files have been corrected.

