# Supplementary material for: Using machine learning to predict student retention from socio-demographic characteristics and app-based engagement metrics
Source: Sci Rep. 2023 Apr 7;13:5705. doi: 10.1038/s41598-023-32484-w (PMC10082180; doi:10.1038/s41598-023-32484-w)

**Supplementary Materials**

Matz SC, Bukow CS, Peters H, Dinu A, Deacons C & Stachl C

Table S1. Overview of all data sets provided by READY Education used for feature extraction.

| Data set | Description |
| --- | --- |
| app_session | List of user's app sessions with all app open and app close times |
| campus_event | List of all campus events with title, description, host type (service, club), host id as well as start time and end time |
| campus_event_attendance | List of all user's attended campus events (whenever users attend an event, they scan an event-specific QR code, which generates the log) with the event id, checkin and checkout time, feedback rating and feedback text |
| campus_wall_comment | List of all user's comments on campus wall posts with the comment text, number of likes received for the comment and time log |
| campus_wall_comment_like | List of all user's likes of comments of campus wall posts with time log |
| campus_wall_post | List of all user's campus wall posts with the post text, number of likes and number of comments received and time log |
| campus_wall_post_like | List of all user's likes of campus wall posts with time log |
| chat_message | List of all user's chat messages with the user id of the receiver and time log |
| clicked_campus_guide_tab | List of all user's clicks on the campus guide tab in the app with time log |
| clicked_community_tab | List of all user's clicks on the community tab in the app with time log |
| clicked_home_tab | List of all user's clicks on the home tab in the app with time log |
|  |  |
| Table S1 (continued) |  |
| Data set | Description |
| clicked_notifications_tab | List of all user's clicks on the notifications tab in the app with time log |
| clicked_profile_tab | List of all user's clicks on the profile tab in the app with time log |
| clicked_tile | List of all user's clicks on tiles with the source coming from and time log |
| download_and_registration | List of all user's times of app download and app registration |
| friend_request | List of all user's friend requests with user id of the receiver and the time the request was sent and the time the request was accepted (unaccepted requests are not included) |
| orientation_event | List of all orientation events with title, description, start time and end time |
| orientation_event_attendance | List of all user's attended orientation events (whenever users attend an event, they scan an event-specific QR code, which generates the log) with the event id, checkin and checkout time, feedback rating and feedback text |
| performed_search | List of all user's performed searches with source coming from and time log |
| service_attendance | List of all user's attended services (whenever a person makes use of a campus service, they can a QR which is always the same for the same service and generates the log) with the name of the service provider, the checkin and checkout time, feedback rating and text |
| social_group_member | List of all user's social group memberships and the time log of joining the group |
| social_group_comment | List of all user's comments on social group posts with the comment text, number of likes received for the comment and time log |
| social_group_comment_like | List of all user's likes of comments of social group posts with time log |
|  |  |
| Table S1 (continued) |  |
| Data set | Description |
| social_group_post | List of all user's social group posts with the post text, number of likes and number of comments received and time log |
| social_group_post_like | List of all user's likes of social group posts with time log |
| user_added_course | List of all user's courses added with course title, description, and time log |
| user_campus_event | List of all user's campus events added to own calendar to follow |
| user_retention_data | List of all Institutional |
| viewed_campus_event | List of all user's campus events viewed in the app with time log |
| viewed_club | List of all user's clubs viewed in the app with time log |
| viewed_user_profile | List of all user's user profiles viewed in the app with time log |

Table S2. *Overview of all features extracted from the raw data of the two universities.*

| Category | Name | Description |
| --- | --- | --- |
| App. eng. | ae_total_app_used_days | Total days app was used |
| App. eng. | ae_total_sess | Total number of app sessions |
| App. eng. | ae_total_sess_dur | Total time (seconds) spent on app sessions in seconds |
| App. eng. | ae_mean_sess_dur | Mean app session duration in seconds |
| App. eng. | ae_sd_sess_dur | SD of app session durations in seconds |
| App. eng. | ae_max_sess_dur | Maximum of app session durations in seconds |
| App. eng. | ae_min_sess_dur | Minimum of app session durations in seconds |
| App. eng. | ae_IQR_sess_dur | Midspread of duration of an app session (50% of app session durations) |
| App. eng. | ae_skew_sess_dur | Skewness of app session durations |
| App. eng. | ae_kurt_sess_dur | Kurtosis of app session durations |
| App. eng. | ae_mean_time_between_sess | Mean time (seconds) between two app sessions |
| App. eng. | ae_sd_time_between_sess | SD of time (seconds) between two app sessions |
| App. eng. | ae_max_time_between_sess | Maximum of time (seconds) between two app sessions |
| App. eng. | ae_min_time_between_sess | Minimum of time (seconds) passed between two app sessions |
| App. eng. | ae_IQR_time_between_sess | Midspread of time (seconds) between two sessions (50% of app session durations) |
| App. eng. | ae_skew_time_between_sess | Skewness of time (seconds) between two app sessions |
| App. eng. | ae_kurt_time_between_sess | Kurtosis of time (seconds) between two app sessions |
| App. eng. | ae_app_before_firstterm | Student used the app before the start of their first term [T/F] |
| Table S2 (continued) | |  |
| Category | Name | Description |
| App. eng. | ae_app_first_winter | Student used the app in the winter break after their first term [T/F] |
| App. eng. | ae_total_app_used_weekdays | Total days app was used during the week (Mon-Fri) |
| App. eng. | ae_total_sess_weekdays | Total number of app sessions during the week (Mon-Fri) |
| App. eng. | ae_total_sess_dur_weekdays | Total time (seconds) spent on app sessions in seconds during the week (Mon-Fri) |
| App. eng. | ae_mean_sess_dur_weekdays | Mean app session duration during the week (Mon-Fri) in seconds |
| App. eng. | ae_sd_sess_dur_weekdays | SD of app session durations during the week (Mon-Fri) in seconds |
| App. eng. | ae_max_sess_dur_weekdays | Maximum of app session durations during the week (Mon-Fri) in seconds |
| App. eng. | ae_min_sess_dur_weekdays | Minimum of app session durations during the week (Mon-Fri) in seconds |
| App. eng. | ae_IQR_sess_dur_weekdays | Midspread duration of an app session (50% of app session durations) during the week (Mon-Fri) |
| App. eng. | ae_skew_sess_dur_weekdays | Skewness of app session durations during the week (Mon-Fri) |
| App. eng. | ae_kurt_sess_dur_weekdays | Kurtosis of app session durations during the week (Mon-Fri) |
| App. eng. | ae_total_app_used_weekend | Total days app was used on weekends |
| App. eng. | ae_total_sess_weekend | Total number of app sessions on weekends |
| App. eng. | ae_total_sess_dur_weekend | Total time (seconds) spent on app sessions on weekends |
| App. eng. | ae_mean_sess_dur_weekend | Mean app session duration on weekends in seconds |
| App. eng. | ae_sd_sess_dur_weekend | SD of app session durations on weekends in seconds |
| Table S2 (continued) | |  |
| Category | Name | Description |
| App. eng. | ae_max_sess_dur_weekend | Maximum of app session durations on weekends in seconds |
| App. eng. | ae_min_sess_dur_weekend | Minimum of app session durations on weekends in seconds |
| App. eng. | ae_IQR_sess_dur_weekend | Midspread duration of an app session (50% of app session durations) on weekends |
| App. eng. | ae_skew_sess_dur_weekend | Skewness of app session durations on weekends |
| App. eng. | ae_kurt_sess_dur_weekend | Kurtosis of app session durations on weekends |
| App. eng. | ae_ratio_total_app_used_weekday_weekend | Ratio of total days app was used on weekdays compared to weekends |
| App. eng. | ae_ratio_total_sess_weekday_to_weekend | Ratio of total number of app sessions on weekdays compared to weekends |
| App. eng. | ae_ratio_total_sess_dur_weekday_to_weekend | Ratio of total time spent on app sessions on weekdays compared to weekends in seconds |
| App. eng. | ae_ratio_mean_sess_dur_weekday_to_weekend | Ratio of mean app session duration on weekdays compared to weekends in seconds |
| App. eng. | ae_ratio_sd_sess_dur_weekday_to_weekend | Ratio of SD app session duration on weekdays compared to weekends in seconds |
| App. eng. | ae_coef_sessdur | Slope coefficient from regression of daily average of session duration on the date, showing change of session duration over time |
| App. eng. | ae_coef_totalsess | Slope coefficient from regression of number of session per day the on date, showing change of number of sessions over time |
| Com. eng. | ce_total_camp_ev | Total number of campus events attended |
| Com. eng. | ce_mean_rat_camp_ev | Mean of campus event ratings |
| Table S2 (continued) | |  |
| Category | Name | Description |
| Com. eng. | ce_sd_rat_camp_ev | SD of campus event ratings |
| Com. eng. | ce_max_rat_camp_ev | Maximum of campus event ratings |
| Com. eng. | ce_min_rat_camp_ev | Minimum of campus event ratings |
| Com. eng. | ce_mean_time_between_ev | Mean time (seconds) between two campus events attended |
| Com. eng. | ce_sd_time_between_ev | SD of time (seconds) between two campus events attended |
| Com. eng. | ce_max_time_between_ev | Maximum of time (seconds) between two campus events attended |
| Com. eng. | ce_min_time_between_ev | Minimum of time (seconds) passed between two campus events attended |
| Com. eng. | ce_IQR_time_between_ev | Midspread of time (seconds) between two campus events attended (50% of campus events) |
| Com. eng. | ce_skew_time_between_ev | Skewness of time (seconds) between two campus events attended |
| Com. eng. | ce_kurt_time_between_ev | Kurtosis of time (seconds) between two campus events attended |
| Com. eng. | ce_total_rat_camp_ev_1star | Total number of 1-star ratings given for campus events |
| Com. eng. | ce_total_rat_camp_ev_2star | Total number of 2-star ratings given for campus events |
| Com. eng. | ce_total_rat_camp_ev_3star | Total number of 3-star ratings given for campus events |
| Com. eng. | ce_total_rat_camp_ev_4star | Total number of 4-star ratings given for campus events |
| Com. eng. | ce_total_rat_camp_ev_5star | Total number of 5-star ratings given for campus events |
| Com. eng. | ce_ratio_1star_to_rest | Ratio of 1-star ratings given to all ratings given for campus events |
| Com. eng. | ce_ratio_2star_to_rest | Ratio of 2-star ratings given to all ratings given for campus events |
| Table S2 (continued) | |  |
| Category | Name | Description |
| Com. eng. | ce_ratio_3star_to_rest | Ratio of 3-star ratings given to all ratings given for campus events |
| Com. eng. | ce_ratio_4star_to_rest | Ratio of 4-star ratings given to all ratings given for campus events |
| Com. eng. | ce_ratio_5star_to_rest | Ratio of 5-star ratings given to all ratings given for campus events |
| Com. eng. | ce_total_post_wl | Total number of wall posts |
| Com. eng. | ce_total_likes_rec_post_wl | Total number of likes received for wall posts |
| Com. eng. | ce_mean_likes_rec_post_wl | Mean of likes received per wall post |
| Com. eng. | ce_sd_likes_rec_post_wl | SD of likes received per wall post |
| Com. eng. | ce_max_likes_rec_post_wl | Maximum of likes received per wall post |
| Com. eng. | ce_min_likes_rec_post_wl | Minimum of likes received per wall post |
| Com. eng. | ce_skew_likes_rec_post_wl | Skewness of likes received per wall post |
| Com. eng. | ce_kurt_likes_rec_post_wl | Kurtosis of likes received per wall post |
| Com. eng. | ce_total_com_rec_post_wl | Total number of comments received for wall post |
| Com. eng. | ce_mean_com_rec_post_wl | Mean of comments received per wall post |
| Com. eng. | ce_sd_com_rec_post_wl | SD of comments received per wall post |
| Com. eng. | ce_max_com_rec_post_wl | Maximum of comments received per wall post |
| Com. eng. | ce_min_com_rec_post_wl | Minimum of comments received per wall post |
| Com. eng. | ce_skew_com_rec_post_wl | Skewness of comments received per wall post |
| Com. eng. | ce_kurt_com_rec_post_wl | Kurtosis of comments received per wall post |
| Com. eng. | ce_mean_time_between_posts_wl | Mean time (seconds) between two wall posts |
| Table S2 (continued) | |  |
| Category | Name | Description |
| Com. eng. | ce_sd_time_between_posts_wl | SD of time (seconds) between two wall posts |
| Com. eng. | ce_max_time_between_posts_wl | Maximum of time (seconds) between two wall posts |
| Com. eng. | ce_min_time_between_posts_wl | Minimum of time (seconds) passed between two wall posts |
| Com. eng. | ce_IQR_time_between_posts_wl | Midspread of time (seconds) between two wall posts (50% of wall posts) |
| Com. eng. | ce_skew_time_between_posts_wl | Skewness of time (seconds) between two wall posts |
| Com. eng. | ce_kurt_time_between_posts_wl | Kurtosis of time (seconds) between two wall posts |
| Com. eng. | ce_ratio_post_posttolike_wl | Ratio of posts to likes received for wall posts |
| Com. eng. | ce_ratio_post_posttocom_wl | Ratio of posts to comments received for wall posts |
| Com. eng. | ce_ratio_likestocom_wl | Ratio of likes received and comments received for wall posts |
| Com. eng. | ce_total_words_post_wl | Total number of words in all wall posts |
| Com. eng. | ce_mean_words_post_wl | Mean number of words per wall post |
| Com. eng. | ce_total_diff_words_post_wl | Total number of different words in all wall posts |
| Com. eng. | ce_mean_diff_words_per_post_wl | Mean number of different words per wall post |
| Com. eng. | ce_ratio_diff_words_to_all_words_post_wl | Ratio of different words to all words used in wall posts |
| Com. eng. | ce_total_sentiment_pos_post_wl | Total number of positive words in all wall posts (acc. to NRC lexicon) |
| Com. eng. | ce_mean_sentiment_pos_per_post_wl | Mean number of positive words per wall post (acc. to NRC lexicon) |
| Com. eng. | ce_total_sentiment_neg_post_wl | Total number of negative words in all wall posts (acc. to NRC lexicon) |
|  |  |  |
| Table S2 (continued) | |  |
| Category | Name | Description |
| Com. eng. | ce_mean_sentiment_neg_per_post_wl | Mean number of negative words per wall post (acc. to NRC lexicon) |
| Com. eng. | ce_net_sentiment_post_wl | Net sentiment (positive - negative) of words used in wall posts (acc. to NRC lexicon) |
| Com. eng. | ce_total_likes_giv_post_wl | Total number of wall post likes given |
| Com. eng. | ce_total_com_post_wl | Total number of wall comments written |
| Com. eng. | ce_total_likes_rec_com_post_wl | Total number of likes received on wall comments |
| Com. eng. | ce_mean_likes_rec_com_post_wl | Mean of likes received per wall comment |
| Com. eng. | ce_sd_likes_rec_com_post_wl | SD of likes received per wall comment |
| Com. eng. | ce_max_likes_rec_com_post_wl | Maximum of likes received per wall comment |
| Com. eng. | ce_min_likes_rec_com_post_wl | Minimum of likes received per wall comment |
| Com. eng. | ce_skew_likes_rec_com_post_wl | Skewness of likes received per wall comment |
| Com. eng. | ce_kurt_likes_rec_com_post_wl | Kurtosis of likes received per wall comment |
| Com. eng. | ce_ratio_com_liketocom_post_wl | Ratio of total wall comments posted and likes received on wall comments |
| Com. eng. | ce_total_words_com_post_wl | Total number of words in all wall post comments |
| Com. eng. | ce_mean_words_com_post_wl | Mean number of words per wall post comment |
| Com. eng. | ce_total_diff_words_com_post_wl | Total number of different words in all wall post comments |
| Com. eng. | ce_mean_diff_words_per_com_post_wl | Mean number of different words per wall post comment |
| Com. eng. | ce_total_sentiment_pos_com_post_wl | Ratio of different words to all words used in wall post comments |
| Table S2 (continued) | |  |
| Category | Name | Description |
| Com. eng. | ce_mean_sentiment_pos_per_com_post_wl | Total number of positive words in all wall post comments (acc. to NRC) |
| Com. eng. | ce_total_sentiment_neg_com_post_wl | Mean number of positive words per wall post comment (acc. to NRC) |
| Com. eng. | ce_mean_sentiment_neg_per_com_post_wl | Total number of negative words in all wall post comments (acc. to NRC) |
| Com. eng. | ce_ratio_diff_words_to_all_words_com_post_wl | Mean number of negative words per wall post comment (acc. to NRC) |
| Com. eng. | ce_net_sentiment_com_post_wl | Net sentiment (positive - negative) of words used in wall post comments (acc. to NRC) |
| Com. eng. | ce_entropy_com_post_wl | Entropy of wall comments written on different posts |
| Com. eng. | ce_total_likes_giv_com_post_wl | Total number of wall comment likes given |
| Com. eng. | ce_total_soc_grp | Total number of social group memberships |
| Com. eng. | ce_total_post_sg | Total number of posts in social group |
| Com. eng. | ce_total_likes_rec_post_sg | Total number of likes received for s.g. posts |
| Com. eng. | ce_mean_likes_rec_post_sg | Mean of likes received per s.g. post |
| Com. eng. | ce_sd_likes_rec_post_sg | SD of likes received per s.g. post |
| Com. eng. | ce_max_likes_rec_post_sg | Maximum of likes received per s.g. post |
| Com. eng. | ce_min_likes_rec_post_sg | Minimum of likes received per s.g. post |
| Com. eng. | ce_skew_likes_rec_post_sg | Skewness of likes received per s.g. post |
| Com. eng. | ce_kurt_likes_rec_post_sg | Kurtosis of likes received per s.g. post |
| Com. eng. | ce_total_com_rec_post_sg | Total number of comments received for s.g. posts |
| Table S2 (continued) | |  |
| Category | Name | Description |
| Com. eng. | ce_mean_com_rec_post_sg | Mean of comments received per s.g. posts |
| Com. eng. | ce_sd_com_rec_post_sg | SD of comments received per s.g. posts |
| Com. eng. | ce_max_com_rec_post_sg | Maximum of comments received per s.g. posts |
| Com. eng. | ce_min_com_rec_post_sg | Minimum of comments received per s.g. posts |
| Com. eng. | ce_skew_com_rec_post_sg | Skewness of comments received per s.g. posts |
| Com. eng. | ce_kurt_com_rec_post_sg | Kurtosis of comments received per s.g. posts |
| Com. eng. | ce_mean_time_between_posts_sg | Mean time (seconds) between two s.g. posts |
| Com. eng. | ce_sd_time_between_posts_sg | SD of time (seconds) between two s.g. posts |
| Com. eng. | ce_max_time_between_posts_sg | Maximum of time (seconds) between two s.g. posts |
| Com. eng. | ce_min_time_between_posts_sg | Minimum of time (seconds) passed between two s.g. posts |
| Com. eng. | ce_IQR_time_between_posts_sg | Midspread of time (seconds) between two s.g. posts |
| Com. eng. | ce_skew_time_between_posts_sg | Skewness of time (seconds) between two s.g. posts |
| Com. eng. | ce_kurt_time_between_posts_sg | Kurtosis of time (seconds) between two s.g. posts |
| Com. eng. | ce_ratio_post_posttolike_sg | Ratio of posts to likes received for s.g. posts |
| Com. eng. | ce_ratio_post_posttocom_sg | Ratio of posts to comments received for s.g. posts |
| Com. eng. | ce_ratio_likestocom_sg | Ratio of likes received and comments received for s.g. posts |
| Com. eng. | ce_total_words_post_sg | Total number of words in all s.g. posts |
| Com. eng. | ce_mean_words_post_sg | Mean number of words per s.g. post |
| Com. eng. | ce_total_diff_words_post_sg | Total number of different words in all s.g. posts |
| Com. eng. | ce_mean_diff_words_per_post_sg | Mean number of different words per s.g. post |
| Table S2 (continued) | |  |
| Category | Name | Description |
| Com. eng. | ce_ratio_diff_words_to_all_words_post_sg | Ratio of different words to all words used in s.g. posts |
| Com. eng. | ce_total_sentiment_pos_post_sg | Total number of positive words in all s.g. posts (acc. to NRC lexicon) |
| Com. eng. | ce_mean_sentiment_pos_per_post_sg | Mean number of positive words per s.g. post (acc. to NRC lexicon) |
| Com. eng. | ce_total_sentiment_neg_post_sg | Total number of negative words in all s.g. posts (acc. to NRC lexicon) |
| Com. eng. | ce_mean_sentiment_neg_per_post_sg | Mean number of negative words per s.g. post (acc. to NRC lexicon) |
| Com. eng. | ce_net_sentiment_post_sg | Net sentiment (positive - negative) of words used in s.g. posts (acc. to NRC lexicon) |
| Com. eng. | ce_total_likes_giv_post_sg | Total number of s.g. post likes given |
| Com. eng. | ce_total_com_post_sg | Total number of s.g comments written |
| Com. eng. | ce_total_likes_rec_com_post_sg | Total number of likes received for s.g. comment |
| Com. eng. | ce_mean_likes_rec_com_post_sg | Mean of likes received per s.g comment |
| Com. eng. | ce_sd_likes_rec_com_post_sg | SD of likes received per s.g comment |
| Com. eng. | ce_max_likes_rec_com_post_sg | Maximum of likes received per s.g comment |
| Com. eng. | ce_min_likes_rec_com_post_sg | Minimum of likes received per s.g comment |
| Com. eng. | ce_skew_likes_rec_com_post_sg | Skewness of likes received per s.g comment |
| Com. eng. | ce_kurt_likes_rec_com_post_sg | Kurtosis of likes received per s.g comment |
| Com. eng. | ce_ratio_com_liketocom_post_sg | Ratio of total s.g. comments posted and likes received on s.g. comments |
| Table S2 (continued) | |  |
| Category | Name | Description |
| Com. eng. | ce_total_words_com_post_sg | Total number of words in all s.g. post comments |
| Com. eng. | ce_mean_words_com_post_sg | Mean number of words per s.g. post comment |
| Com. eng. | ce_total_diff_words_com_post_sg | Total number of different words in all s.g. post comments |
| Com. eng. | ce_mean_diff_words_per_com_post_sg | Mean number of different words per s.g. post comment |
| Com. eng. | ce_total_sentiment_pos_com_post_sg | Ratio of different words to all words used in s.g. post comments |
| Com. eng. | ce_mean_sentiment_pos_per_com_post_sg | Total number of positive words in all s.g. post comments (acc. to NRC lexicon) |
| Com. eng. | ce_total_sentiment_neg_com_post_sg | Mean number of positive words per s.g. post comment (acc. to NRC lexicon) |
| Com. eng. | ce_mean_sentiment_neg_per_com_post_sg | Total number of negative words in all s.g. post comments (acc. to NRC lexicon) |
| Com. eng. | ce_ratio_diff_words_to_all_words_com_post_sg | Mean number of negative words per s.g. post comment (acc. to NRC lexicon) |
| Com. eng. | ce_net_sentiment_com_post_sg | Net sentiment (positive - negative) of words used in s.g. post comments (acc. to NRC lexicon) |
| Com. eng. | ce_entropy_com_post_sg | Entropy of s.g. comments written on different posts |
| Com. eng. | ce_total_likes_giv_com_post_sg | Total number of s.g. comment likes given |
| Com. eng. | ce_ratio_posttocom_sg | Ratio of posts to comments in social groups |
| Com. eng. | ce_total_posts | Total number of posts (in social groups and on campus wall) |
| Com. eng. | ce_total_coms | Total number of comments on posts (in social groups and on campus wall) |
| Table S2 (continued) | |  |
| Category | Name | Description |
| Com. eng. | ce_ratio_posttocom_all | Ratio of posts to comments written on wall and in social groups in total |
| Com. eng. | ce_total_messages | Total number of messages |
| Com. eng. | ce_total_chat_msg_sent | Total number of messages sent |
| Com. eng. | ce_total_chat_msg_rec | Total number of messages received |
| Com. eng. | ce_total_addressees | Total number of people a student sent messages to |
| Com. eng. | ce_total_senders | Total number of people a student received messages from |
| Com. eng. | ce_ratio_chat_msg_addtosend | Ratio of message addressees to senders of a person |
| Com. eng. | ce_ratio_chat_msg_rectosent | Ratio of messages received to sent |
| Com. eng. | ce_mean_time_between_msg_sent | Mean time between sending two messages |
| Com. eng. | ce_sd_time_between_msg_sent | SD of time between sending two messages |
| Com. eng. | ce_max_time_between_msg_sent | Maximum of time between sending two messages |
| Com. eng. | ce_min_time_between_msg_sent | Minimum of time passed between sending two messages |
| Com. eng. | ce_IQR_time_between_msg_sent | Midspread of time (seconds) between sending two messages (50% of messages sent) |
| Com. eng. | ce_skew_time_between_msg_sent | Skewness of time between sending two messages |
| Com. eng. | ce_kurt_time_between_msg_sent | Kurtosis of time between sending two messages |
| Com. eng. | ce_mean_time_between_msg_rec | Mean time between receiving two messages |
| Com. eng. | ce_sd_time_between_msg_rec | SD of time between receiving two messages |
| Com. eng. | ce_max_time_between_msg_rec | Maximum of time between receiving two messages |
| Com. eng. | ce_min_time_between_msg_rec | Minimum of time passed between receiving two messages |
| Table S2 (continued) | |  |
| Category | Name | Description |
| Com. eng. | ce_IQR_time_between_msg_rec | Midspread of time (seconds) between receiving two messages (50% of messages received) |
| Com. eng. | ce_skew_time_between_msg_rec | Skewness of time between receiving two messages |
| Com. eng. | ce_kurt_time_between_msg_rec | Kurtosis of time between receiving two messages |
| Com. eng. | ce_entropy_addressees | Entropy of different addressees in chat |
| Com. eng. | ce_entropy_senders | Entropy of different senders in chat |
| Com. eng. | ce_perc_overlap_contact_weekend_weekday | Percentage of contacts messaged both on weekends and weekdays |
| Com. eng. | ce_coef_mes_sent | Slope coefficient from regression of number of messages sent per day on the date, showing change of number of messages sent over time |
| Com. eng. | ce_coef_mes_rec | Slope coefficient from regression of number of messages received per day on the date, showing change of number of messages received over time |
| App. eng. | ae_total_click_cg | Total number of clicks on Campus Guide tab |
| App. eng. | ae_total_days_click_cg | Total number of days with click(s) on Campus Guide tab |
| App. eng. | ae_coef_click_cg | Slope coefficient from regression of number of clicks on Campus Guide tab per day on the date, showing change of number of clicks over time |
| App. eng. | ae_total_click_cmnty | Total number of clicks on Community tab |
| App. eng. | ae_total_days_click_cmnty | Total number of days with click(s) on Community tab |
| App. eng. | ae_coef_click_cmnty | Slope coefficient from regression of number of clicks on Community tab per day on the date, showing change of number of clicks over time |
| Table S2 (continued) | |  |
| Category | Name | Description |
| App. eng. | ae_total_click_hm | Total number of clicks on Home tab |
| App. eng. | ae_total_days_click_hm | Total number of days with click(s) on Home tab |
| App. eng. | ae_total_click_not | Total number of clicks on Notifications tab |
| App. eng. | ae_total_days_click_not | Total number of days with click(s) on Notifications tab |
| App. eng. | ae_coef_click_not | Slope coefficient from regression of number of clicks on notifications tab per day on the date, showing change of number of clicks over time |
| App. eng. | ae_total_click_prfl | Total number clicks on Profile tab |
| App. eng. | ae_total_days_click_prfl | Total number of days with click(s) on Profile tab |
| App. eng. | ae_coef_click_prfl | Slope coefficient from regression of number of clicks on profile tab per day on the date, showing change of number of clicks over time |
| App. eng. | ae_total_click_tiles | Total number clicks on tiles |
| App. eng. | ae_total_days_click_tiles | Total number of days with click(s) on tiles |
| App. eng. | ae_total_click_diff_tiles | Number of different tiles clicked |
| App. eng. | ae_total_diff_click_sour | Number of different sources coming from |
| App. eng. | ae_coef_click_tiles | Slope coefficient from regression of number of clicks on tiles per day on the date, showing change of number of clicks over time |
| App. eng. | ae_time_to_reg | Time elapse (seconds) between download and registration |
| Com. eng. | ce_total_friends | Total number of friends |
| Com. eng. | ce_total_frnd_req_sent | Total number of requests sent |
| Table S2 (continued) | |  |
| Category | Name | Description |
| Com. eng. | ce_total_time_wait | Total time (seconds) waited for acceptance of a friend request |
| Com. eng. | ce_mean_time_wait | Mean time (seconds) waited for acceptance per friend request |
| Com. eng. | ce_sd_time_wait | SD time (seconds) waited for acceptance per friend request |
| Com. eng. | ce_max_time_wait | Maximum time (seconds) waited for acceptance per friend request |
| Com. eng. | ce_min_time_wait | Minimum time (seconds) waited for acceptance per friend request |
| Com. eng. | ce_skew_time_wait | Skewness of time (seconds) waited for acceptance per friend request |
| Com. eng. | ce_kurt_time_wait | Kurtosis of time (seconds) waited for acceptance per friend request |
| Com. eng. | ce_total_frnd_req_rec | Total number of friend requests received |
| Com. eng. | ce_total_time_acc | Total time (seconds) taken to accept a friend request |
| Com. eng. | ce_mean_time_acc | Mean time (seconds) taken to accept a friend request |
| Com. eng. | ce_sd_time_acc | SD of time (seconds) taken to accept a friend request |
| Com. eng. | ce_max_time_acc | Maximum time (seconds) taken to accept a friend request |
| Com. eng. | ce_min_time_acc | Minimum time (seconds) taken to accept a friend request |
| Com. eng. | ce_skew_time_acc | Skewness of time (seconds) taken to accept a friend request |
| Com. eng. | ce_kurt_time_acc | Kurtosis of time (seconds) taken to accept a friend request |
| Com. eng. | ce_ratio_frnd_req_rectosent | Ratio of friend requests received to requests sent |
| Com. eng. | ce_ratio_time_waittoacc | Ratio of time waited for friend requests being accepted and accepting requests |
| Com. eng. | ce_mean_rat_or_ev | Mean rating of orientation events |
| Table S2 (continued) | |  |
| Category | Name | Description |
| Com. eng. | ce_sd_rat_or_ev | SD rating of orientation events |
| Com. eng. | ce_max_rat_or_ev | Maximum rating of orientation events |
| Com. eng. | ce_min_rat_or_ev | Minimum rating of orientation events |
| App. eng. | ae_total_search | Total number of performed searches |
| App. eng. | ae_total_diff_search_source | Total number of different sources when searching |
| App. eng. | ae_total_source_home | Total number of searches performed from Home |
| App. eng. | ae_total_source_guide | Total number of searches performed from Campus Guide |
| Com. eng. | ce_total_serv | Total number of services attended |
| Com. eng. | ce_mean_rat_serv | Mean feedback rating services |
| Com. eng. | ce_sd_rat_serv | SD of feedback rating services |
| Com. eng. | ce_max_rat_serv | Maximum feedback rating services |
| Com. eng. | ce_min_rat_serv | Minimum feedback rating services |
| Com. eng. | ce_mean_time_between_serv | Mean time between two services attended |
| Com. eng. | ce_sd_time_between_serv | SD of time between two services attended |
| Com. eng. | ce_max_time_between_serv | Maximum of time between two services attended |
| Com. eng. | ce_min_time_between_serv | Minimum of time passed between two services attended |
| Com. eng. | ce_IQR_time_between_serv | Midspread of time (seconds) between two services attended (50% of service attendance) |
| Com. eng. | ce_skew_time_between_serv | Skewness of time between two services attended |
| Table S2 (continued) | |  |
| Category | Name | Description |
| Com. eng. | ce_kurt_time_between_serv | Kurtosis of time between two services attended |
| Com. eng. | ce_entropy_serv_att | Entropy of different services attended |
| App. eng. | ae_total_cour_add | Total number of courses added |
| App. eng. | ae_total_ev_followed | Total number of events added to calendar to follow |
| App. eng. | ae_total_view_ev | Total number of in-app views of event(s) |
| App. eng. | ae_total_days_view_ev | Total number of days of in-app views of event(s) |
| App. eng. | ae_coef_view_campev | Slope coefficient from regression of number of views of campus events per day on the date, showing change of number of clicks over time |
| App. eng. | ae_total_view_club | Total number of in-app views of club(s) |
| App. eng. | ae_total_days_view_club | Total number of days of in-app views of club(s) |
| App. eng. | ae_coef_view_club | Slope coefficient from regression of number of views of clubs per day on the date, showing change of number of clicks over time |
| App. eng. | ae_total_view_prfl | Total number of in-app views of profile(s) |
| App. eng. | ae_total_days_view_prfl | Total number of days of in-app views of profile(s) |
| Institutional | inst_student_degree_type | Student's degree type |
| Institutional | inst_cumulative_gpa | Student's cumulative GPA score |
| Institutional | inst_act_score | Student's ACT score |
|  |  |  |
| Table S2 (continued) | |  |
| Category | Name | Description |
| Institutional | inst_high_school_gpa | Student's high school GPA score |
| Institutional | inst_gender | Student's gender |
| Institutional | inst_living_in_residence | Student's housing situation (on- or off-campus) |
| Institutional | inst_athlete | Student's athlete status |
| Institutional | inst_pell_eligibile | Student's pell eligibility |
| Institutional | inst_ethnicity | Student's ethnicity |
| Institutional | inst_in_state | Student's state of origin (from the same state the university is in or out of state) |
| Institutional | inst_program_status | Student's mode of studying (fulltime vs. parttime) |
| Institutional | inst_spiritual_identity | Student's spiritual identity |
| Institutional | inst_international_student | Student's citizen status (US citizens and students with a permanent resident status are considered domestic, others as international) |
| Com. eng. | ce_ratio_msgrec_to_postwl | Ratio of messages received to posts made on campus wall |
| Com. eng. | ce_ratio_msgsent_to_postwl | Ratio of messages sent to posts made on campus wall |
| Com. eng. | ce_ratio_msg_to_postwl | Ratio of total of messages to posts made on campus wall |
| Table S2 (continued) | |  |
| Category | Name | Description |
| Com. eng. | ce_ratio_msgrec_to_postwlcom | Ratio of messages received to comments made on posts on campus wall |
| Com. eng. | ce_ratio_msgsent_to_postwlcom | Ratio of messages sent to comments made on posts on campus wall |
| Com. eng. | ce_ratio_msg_to_postwlcom | Ratio of total of messages to comments made on posts on campus wall |
| Com. eng. | ce_ratio_msgrec_to_postsg | Ratio of messages received to posts made in s.g. |
| Com. eng. | ce_ratio_msgsent_to_postsg | Ratio of messages sent to posts made in s.g. |
| Com. eng. | ce_ratio_msg_to_postsg | Ratio of total of messages to posts made in s.g. |
| Com. eng. | ce_ratio_msgrec_to_postsgcom | Ratio of messages received to comments made on posts in s.g. |
| Com. eng. | ce_ratio_msgsent_to_postsgcom | Ratio of messages sent to comments made on posts in s.g. |
| Com. eng. | ce_ratio_msg_to_postsgcom | Ratio of total of messages to comments made on posts in s.g. |
| Com. eng. | ce_ratio_frndrec_to_postwl | Ratio of friend requests received to posts made on campus wall |
| Com. eng. | ce_ratio_frndsent_to_postwl | Ratio of friend requests sent to posts made on campus wall |
| Com. eng. | ce_ratio_frnd_to_postwl | Ratio of total of friends to posts made on campus wall |
| Com. eng. | ce_ratio_frndrec_to_postwlcom | Ratio of friend requests received to comments made on posts on campus wall |
| Com. eng. | ce_ratio_frndsent_to_postwlcom | Ratio of friend requests sent to comments made on posts on campus wall |
| Com. eng. | ce_ratio_frnd_to_postwlcom | Ratio of total of friends to comments made on posts on campus wall |
| Com. eng. | ce_ratio_frndrec_to_msgrec | Ratio of friend requests received to messages received |
| Table S2 (continued) | |  |
| Category | Name | Description |
| Com. eng. | ce_ratio_frndrec_to_msgsent | Ratio of friend requests received to messages sent |
| Com. eng. | ce_ratio_frndsent_to_msgrec | Ratio of friend requests sent to messages received |
| Com. eng. | ce_ratio_frndsent_to_msgsent | Ratio of friend requests sent to messages sent |
| Com. eng. | ce_ratio_frnd_to_msg | Ratio of total number of friends and total number of messages |
| Com. eng. | ce_reg_to_frndreqsent | Time elapse (seconds) between registration and first friend request sent |
| Com. eng. | ce_reg_to_sg_mbr | Time elapse (seconds) between registration and first social group joined |
| Com. eng. | ce_reg_to_camp_ev_att | Time elapse (seconds) between registration and first campus event attended |
| Com. eng. | ce_camp_ev_att_to_frndreqsent | Time elapse (seconds) between first campus event attended and first friend request sent |
| Com. eng. | ce_sg_mbr_to_frndreqsent | Time elapse (seconds) between first group joined and first friend request sent |
| Com. eng. | ce_average_timediff_view_to_frndreq | Average time elapse (seconds) between viewing a user profile in the app and sending a friend request |
| Com. eng. | ce_average_timediff_view_to_goev | Average time elapse (seconds) between viewing an event in the app and going to the event |
| Com. eng. | ce_average_timediff_view_to_goclub | Average time elapse (seconds) between viewing a club in the app and going to an event from that club |
| Com. eng. | ce_total_ev_followed_att | Total number of campus events added to calendar and attended afterwards |
|  |  |  |
| Table S2 (continued) | |  |
| Category | Name | Description |
| Com. eng. | ce_average_timediff_follow_to_goev | Average time elapse (seconds) between adding a campus event to the calendar in the app and going to the same event (if user went to the event at all) |
| Com. eng. | ce_total_ev | Total number of events attended (campus, orientation and services) |
| Com. eng. | ce_total_ev_serv | Total number of campus events and services made use of |
| Com. eng. | ce_mean_rat_ev_serv | Mean rating of campus events and services |
| Com. eng. | ce_sd_rat_ev_serv | SD of rating of campus events and services |
| Com. eng. | ce_total_camp_ev_type_serv | Total campus events attended of hosted by a service |
| Com. eng. | ce_total_camp_ev_type_club | Total campus events attended of hosted by a club |
| Com. eng. | ce_ratio_camp_ev_type_serv_to_club | Ratio of campus events attended hosted by a service to hosted by club |
| Com. eng. | ce_total_camp_ev_title_or | Total number of campus events attended from service or club called "orientation" |
| Com. eng. | ce_total_friends_at_events | Total number of friends that attended a campus event with a user |
| Com. eng. | ce_mean_friends_at_events | Mean of number of friends that attended a campus event with a user |
| Com. eng. | ce_sd_friends_at_events | SD of number of friends that attended a campus event with a user |
| Com. eng. | ce_max_friends_at_events | Maximum number of friends that attended a campus event with a user |
| Com. eng. | ce_min_friends_at_events | Minimum number of friends that attended a campus event with a user |
| Table S2 (continued) | |  |
| Category | Name | Description |
| Com. eng. | ce_skew_friends_at_events | Skewness of number of friends that attended a campus event together with a user |
| Com. eng. | ce_kurt_friends_at_events | Kurtosis of number of friends that attended a campus event together with a user |
| Com. eng. | ce_total_friends_at_services | Total number of friends that attended a service together with a user |
| Com. eng. | ce_mean_friends_at_services | Mean of number of friends that attended a service together with a user |
| Com. eng. | ce_sd_friends_at_services | SD of number of friends that attended a service together with a user |
| Com. eng. | ce_max_friends_at_services | Maximum number of friends that attended a service together with a user |
| Com. eng. | ce_min_friends_at_services | Minimum number of friends that attended a service together with a user |
| Com. eng. | ce_skew_friends_at_services | Skewness of number of friends that attended a service together with a user |
| Com. eng. | ce_kurt_friends_at_services | Kurtosis of number of friends that attended a service together with a user |
| Com. eng. | ce_total_friends_messaged | Total number of friends messages were exchanged with |
| Com. eng. | ce_total_nofriends_messaged | Total number of non-friends messages were exchanged with |
| Com. eng. | ce_total_msg_to_friends | Total number of messages somebody sent to their friends |
| Com. eng. | ce_mean_msg_to_friends | Mean number of messages somebody sent to their friends |
| Com. eng. | ce_sd_msg_to_friends | SD of number of messages somebody sent to their friends |
| Table S2 (continued) | |  |
| Category | Name | Description |
| Com. eng. | ce_max_msg_to_friends | Maximum number of messages somebody sent to their friends |
| Com. eng. | ce_min_msg_to_friends | Minimum number of messages somebody sent to their friends |
| Com. eng. | ce_skew_msg_to_friends | Skewness of the number of messages somebody sent to their friends |
| Com. eng. | ce_kurt_msg_to_friends | Kurtosis of the number of messages somebody sent to their friends |
| Com. eng. | ce_total_msg_to_Nofriends | Total number of messages somebody sent to non-friends |
| Com. eng. | ce_mean_msg_to_Nofriends | Mean number of messages somebody sent to non-friends |
| Com. eng. | ce_sd_msg_to_Nofriends | SD of number of messages somebody sent to non-friends |
| Com. eng. | ce_max_msg_to_Nofriends | Maximum number of messages somebody sent to non-friends |
| Com. eng. | ce_min_msg_to_Nofriends | Minimum number of messages somebody sent to non-friends |
| Com. eng. | ce_skew_msg_to_Nofriends | Skewness of the number of messages somebody sent to non-friends |
| Com. eng. | ce_kurt_msg_to_Nofriends | Kurtosis of the number of messages somebody sent to non-friends |
| Com. eng. | ce_ratio_msgtofrnd_to_msgtoNofrnd | Ratio of total number of messages sent to friends to messages sent to non-friends |
| Com. eng. | ce_total_friends_gottenmsg | Total number of friends messages were received from |
| Com. eng. | ce_total_nofriends_gottenmsg | Total number of non-friends messages were received from |
| Com. eng. | ce_total_msg_from_friends | Total number of messages somebody received from their friends |
| Com. eng. | ce_mean_msg_from_friends | Mean number of messages somebody received from their friends |
| Table S2 (continued) | |  |
| Category | Name | Description |
| Com. eng. | ce_sd_msg_from_friends | SD of number of messages somebody received from their friends |
| Com. eng. | ce_max_msg_from_friends | Maximum number of messages somebody received from their friends |
| Com. eng. | ce_min_msg_from_friends | Minimum number of messages somebody received from their friends |
| Com. eng. | ce_skew_msg_from_friends | Skewness of the number of messages somebody received from their friends |
| Com. eng. | ce_kurt_msg_from_friends | Kurtosis of the number of messages somebody received from their friends |
| Com. eng. | ce_total_msg_from_Nofriends | Total number of messages somebody received from non-friends |
| Com. eng. | ce_mean_msg_from_Nofriends | Mean number of messages somebody received from non-friends |
| Com. eng. | ce_sd_msg_from_Nofriends | SD of number of messages somebody received from non-friends |
| Com. eng. | ce_max_msg_from_Nofriends | Maximum number of messages somebody received from non-friends |
| Com. eng. | ce_min_msg_from_Nofriends | Minimum number of messages somebody received from non-friends |
| Com. eng. | ce_skew_msg_from_Nofriends | Skewness of the number of messages somebody received from non-friends |
| Com. eng. | ce_kurt_msg_from_Nofriends | Kurtosis of the number of messages somebody received from non-friends |
| Com. eng. | ce_ratio_msgfromfrnd_to_msgfromNofrnd | Ratio of total number of messages received from friends to messages received from non-friends |
| Com. eng. | ce_ratio_msgfromfrnd_to_msgfromNofrnd | Ratio of total number of messages received from friends to messages received from non-friends |
| Table S2 (continued) | |  |
| Category | Name | Description |
| Com. eng. | ce_mean_comSocGrpMembrs | Mean number of social group co-members |
| Com. eng. | ce_sd_comSocGrpMembrs | SD of number of social group co-members |
| Com. eng. | ce_max_comSocGrpMembrs | Maximum number of social group co-members |
| Com. eng. | ce_min_comSocGrpMembrs | Minimum number of social group co-members |
| Com. eng. | ce_skew_comSocGrpMembrs | Skewness of the number of social group co-members |
| Com. eng. | ce_kurt_comSocGrpMembrs | Kurtosis of the number of social group co-members |
| Com. eng. | ce_total_comembers_at_events | Total number of co-members of a social group that attended a campus event together with a user |
| Com. eng. | ce_mean_comembers_at_events | Mean of number of co-members of a social group that attended a campus event together with a user |
| Com. eng. | ce_sd_comembers_at_events | SD of number of co-members of a social group that attended a campus event together with a user |
| Com. eng. | ce_max_comembers_at_events | Maximum number of co-members of a social group that attended a campus event together with a user |
| Com. eng. | ce_min_comembers_at_events | Minimum number of co-members of a social group that attended a campus event together with a user |
| Com. eng. | ce_skew_comembers_at_events | Skewness of number of co-members of a social group that attended a campus event together with a user |
| Com. eng. | ce_kurt_comembers_at_events | Kurtosis of number of co-members of a social group that attended a campus event together with a user |
| Com. eng. | ce_total_comembers_at_services | Total number of co-members of a social group that attended a service together with a user |
| Table S2 (continued) | |  |
| Category | Name | Description |
| Com. eng. | ce_mean_comembers_at_services | Mean of number of co-members of a social group that attended a service together with a user |
| Com. eng. | ce_sd_comembers_at_services | SD of number of co-members of a social group that attended a service together with a user |
| Com. eng. | ce_max_comembers_at_services | Maximum number of co-members of a social group that attended a service together with a user |
| Com. eng. | ce_min_comembers_at_services | Minimum number of co-members of a social group that attended a service together with a user |
| Com. eng. | ce_skew_comembers_at_services | Skewness of number of co-members of a social group that attended a service together with a user |
| Com. eng. | ce_kurt_comembers_at_services | Kurtosis of number of co-members of a social group that attended a service together with a user |
| Com. eng. | ce_total_words | Total number of words over all comments, posts and feedback written |
| Com. eng. | ce_total_different_words | Total number of different words over all comments, posts, and feedback written |
| Com. eng. | ce_total_words_sentiment_pos | Total number of positive words over all comments, posts, and feedback written (acc. to NRC lexicon) |
| Com. eng. | ce_total_words_sentiment_neg | Total number of negative words over all comments, posts, and feedback written (acc. to NRC lexicon) |
| Com. eng. | ce_total_words_sentiment_joy | Total number of words classified as joy over all comments, posts, and feedback written (acc. to NRC lexicon) |
|  |  |  |
| Table S2 (continued) | |  |
| Category | Name | Description |
| Com. eng. | ce_total_words_sentiment_anger | Total number of words classified as anger over all comments, posts, and feedback written (acc. to NRC lexicon) |
| Com. eng. | ce_total_words_sentiment_anticipation | Total number of words classified as anticipation over all comments, posts, and feedback written (acc. to NRC lexicon) |
| Com. eng. | ce_total_words_sentiment_disgust | Total number of words classified as disgust over all comments, posts, and feedback written (acc. to NRC lexicon) |
| Com. eng. | ce_total_words_sentiment_fear | Total number of words classified as fear over all comments, posts, and feedback written (acc. to NRC lexicon) |
| Com. eng. | ce_total_words_sentiment_sadness | Total number of words classified as sadness over all comments, posts, and feedback written (acc. to NRC lexicon) |
| Com. eng. | ce_total_words_sentiment_surprise | Total number of words classified as surprise over all comments, posts, and feedback written (acc. to NRC lexicon) |
| Com. eng. | ce_total_words_sentiment_trust | Total number of words classified as trust over all comments, posts, and feedback written (acc. to NRC lexicon) |
| Com. eng. | ce_ratio_diff_words_to_all_words_all | Ratio of different words to all words over all comments, posts, and feedback written |
| Com. eng. | ce_net_sentiment_all | Net sentiment (positive - negative) of words over all comments, posts, and feedback written |
| Com. eng. | ce_total_active_days | Total days with action (active use of the app or registering at an event or service with the app) |
| Com. eng. | ce_total_actions | Total number of actions (active use of the app or registering at an event or service with the app) |
| Table S2 (continued) | |  |
| Category | Name | Description |
| Com. eng. | ce_mean_time_between_actions | Mean time between two actions (active use of the app or registering at an event or service with the app) in seconds |
| Com. eng. | ce_sd_time_between_actions | SD of time between two actions (active use of the app or registering at an event or service with the app) in seconds |
| Com. eng. | ce_max_time_between_actions | Maximum of time between two actions (active use of the app or registering at an event or service with the app) in seconds |
| Com. eng. | ce_IQR_time_between_actions | Midspread of time between two actions (active use of the app or registering at an event or service with the app; 50% of actions) in seconds |
| Com. eng. | ce_skew_time_between_actions | Skewness of time between two actions (active use of the app or registering at an event or service with the app) in seconds |
| Com. eng. | ce_kurt_time_between_actions | Kurtosis of time between two actions (active use of the app or registering at an event or service with the app) in seconds |
| Com. eng. | ce_total_active_weekdays | Total days with action on weekdays (active use of the app or registering at an event or service with the app) |
| Com. eng. | ce_total_actions_weekdays | Total number of actions on weekdays (active use of the app or registering at an event or service with the app) |
| Com. eng. | ce_mean_time_between_actions_weekdays | Mean time between two actions on weekdays (active use of the app or registering at an event or service with the app) in seconds |
| Com. eng. | ce_sd_time_between_actions_weekdays | SD of time between two actions on weekdays (active use of the app or registering at an event or service with the app) in seconds |
|  |  |  |
| Table S2 (continued) | |  |
| Category | Name | Description |
| Com. eng. | ce_max_time_between_actions_weekdays | Maximum of time between two actions on weekdays (active use of the app or registering at an event or service with the app) in seconds |
| Com. eng. | ce_IQR_time_between_actions_weekdays | Midspread of time between two actions on weekdays (active use of the app or registering at an event or service with the app; 50% of actions) in seconds |
| Com. eng. | ce_skew_time_between_actions_weekdays | Skewness of time between two actions on weekdays (active use of the app or registering at an event or service with the app) in seconds |
| Com. eng. | ce_kurt_time_between_actions_weekdays | Kurtosis of time between two actions on weekdays (active use of the app or registering at an event or service with the app) in seconds |
| Com. eng. | ce_total_active_weekend | Total days with action on weekends (active use of the app or registering at an event or service with the app) |
| Com. eng. | ce_total_actions_weekend | Total number of actions on weekends (active use of the app or registering at an event or service with the app) |
| Com. eng. | ce_mean_time_between_actions_weekend | Mean time between two actions on weekends (active use of the app or registering at an event or service with the app) in seconds |
| Com. eng. | ce_sd_time_between_actions_weekend | SD of time between two actions on weekends (active use of the app or registering at an event or service with the app) in seconds |
| Com. eng. | ce_max_time_between_actions_weekend | Maximum of time between two actions on weekends (active use of the app or registering at an event or service with the app) in seconds |
| Com. eng. | ce_IQR_time_between_actions_weekend | Midspread of time between two actions on weekends (active use of the app or registering at an event or service with the app; 50% of actions) in seconds |
| Table S2 (continued) | |  |
| Category | Name | Description |
| Com. eng. | ce_skew_time_between_actions_weekend | Skewness of time between two actions on weekends (active use of the app or registering at an event or service with the app) in seconds |
| Com. eng. | ce_kurt_time_between_actions_weekend | Kurtosis of time between two actions on weekends (active use of the app or registering at an event or service with the app) in seconds |
| Com. eng. | ce_ratio_active_days_weekday_to_weekend | Ratio of total number of active weekdays to active days on the weekend (active use of the app or registering at an event or service with the app) |
| Com. eng. | ce_ratio_total_actions_weekday_to_weekend | Ratio of total number of actions on weekdays to actions on the weekend (active use of the app or registering at an event or service with the app) |
| Com. eng. | ce_ratio_mean_time_between_actions_weekday_to_weekend | Ratio of mean time between actions on weekdays to actions on the weekend (active use of the app or registering at an event or service with the app) in seconds |
| Com. eng. | ce_coef_actions | Slope coefficient from regression of number of actions per day on the date, showing change of number of actions over time |
| Network | net_dm_in_degree_cent | In-degree centrality of a person’s node in social network graph constructed from direct message exchanges |
| Network | net_dm_out_degree_cent | Out-degree centrality of a person’s node in social network graph constructed from direct message exchanges |
| Network | net_dm_eigen_centrality | Eigenvector centrality of a person’s node in social network graph constructed from direct message exchanges |
| Network | net_dm_betweenness | Betweenness centrality of a person’s node in social network graph constructed from direct message exchanges |
| Network | net_dm_closeness | Closeness centrality of a person’s node in social network graph constructed from direct message exchanges |
| Network | net_friend_out_degree_cent | In-degree centrality of a person’s node in social network constructed from friendship graph |
| Network | net_friend_in_degree_cent | Out-degree centrality of a person’s node in social network constructed from friendship graph |
| Network | net_friend_eigen_centrality | Eigenvector centrality of a person’s node in social network constructed from friendship graph |
| Network | net_friend_betweenness | Betweenness centrality of a person’s node in social network constructed from direct message exchanges |
| Network | net_friend_closeness | Closeness centrality of a person’s node in social network constructed from friendship graph |

*Note.* App eng. = App engagement, Com. eng. = Community engagement. “Weekdays” stands for Monday, 00:00 – Friday 17:59, “weekends” stands for Friday, 18:00 – Sunday 23:59.

Table S3. *Confusion matrices (RQ1)*

1. Institutional data

| Uni | Elastic Net | Random Forest |
| --- | --- | --- |
| 1 | \|  \|  \| Pred. \| \| \| --- \| --- \| --- \| --- \| \|  \|  \| Cont. \| Trans. \| \| Obs. \| Cont. \| 253 \| 106 \| \| Trans. \| 49 \| 68 \| | \|  \|  \| Pred. \| \| \| --- \| --- \| --- \| --- \| \|  \|  \| Cont. \| Trans. \| \| Obs. \| Cont. \| 238 \| 121 \| \| Trans. \| 39 \| 78 \| |
| 2 | \|  \|  \| Pred. \| \| \| --- \| --- \| --- \| --- \| \|  \|  \| Cont. \| Trans. \| \| Obs. \| Cont. \| 1421 \| 272 \| \| Trans. \| 167 \| 150 \| | \|  \|  \| Pred. \| \| \| --- \| --- \| --- \| --- \| \|  \|  \| Cont. \| Trans. \| \| Obs. \| Cont. \| 1317 \| 376 \| \| Trans. \| 159 \| 158 \| |
| 3 | \|  \|  \| Pred. \| \| \| --- \| --- \| --- \| --- \| \|  \|  \| Cont. \| Trans. \| \| Obs. \| Cont. \| 1886 \| 258 \| \| Trans. \| 200 \| 203 \| | \|  \|  \| Pred. \| \| \| --- \| --- \| --- \| --- \| \|  \|  \| Cont. \| Trans. \| \| Obs. \| Cont. \| 1828 \| 316 \| \| Trans. \| 199 \| 204 \| |
| 4 | \|  \|  \| Pred. \| \| \| --- \| --- \| --- \| --- \| \|  \|  \| Cont. \| Trans. \| \| Obs. \| Cont. \| 13236 \| 27233 \| \| Trans. \| 849 \| 3744 \| | \|  \|  \| Pred. \| \| \| --- \| --- \| --- \| --- \| \|  \|  \| Cont. \| Trans. \| \| Obs. \| Cont. \| 31752 \| 8717 \| \| Trans. \| 1920 \| 2673 \| |

Note. Obs. = observed classes, Pred. = predicted classes. Cont. = Continued. Trans. = Transferred

1. Behavioral engagement data

| Uni | Elastic Net | Random Forest |
| --- | --- | --- |
| 1 | \|  \|  \| Pred. \| \| \| --- \| --- \| --- \| --- \| \|  \|  \| Cont. \| Trans. \| \| Obs. \| Cont. \| 185 \| 174 \| \| Trans. \| 34 \| 83 \| | \|  \|  \| Pred. \| \| \| --- \| --- \| --- \| --- \| \|  \|  \| Cont. \| Trans. \| \| Obs. \| Cont. \| 322 \| 37 \| \| Trans. \| 48 \| 69 \| |
| 2 | \|  \|  \| Pred. \| \| \| --- \| --- \| --- \| --- \| \|  \|  \| Cont. \| Trans. \| \| Obs. \| Cont. \| 961 \| 732 \| \| Trans. \| 102 \| 215 \| | \|  \|  \| Pred. \| \| \| --- \| --- \| --- \| --- \| \|  \|  \| Cont. \| Trans. \| \| Obs. \| Cont. \| 1340 \| 353 \| \| Trans. \| 189 \| 128 \| |
| 3 | \|  \|  \| Pred. \| \| \| --- \| --- \| --- \| --- \| \|  \|  \| Cont. \| Trans. \| \| Obs. \| Cont. \| 1194 \| 950 \| \| Trans. \| 124 \| 279 \| | \|  \|  \| Pred. \| \| \| --- \| --- \| --- \| --- \| \|  \|  \| Cont. \| Trans. \| \| Obs. \| Cont. \| 1340 \| 353 \| \| Trans. \| 189 \| 128 \| |
| 4 | \|  \|  \| Pred. \| \| \| --- \| --- \| --- \| --- \| \|  \|  \| Cont. \| Trans. \| \| Obs. \| Cont. \| 19138 \| 21331 \| \| Trans. \| 1503 \| 3090 \| | \|  \|  \| Pred. \| \| \| --- \| --- \| --- \| --- \| \|  \|  \| Cont. \| Trans. \| \| Obs. \| Cont. \| 22648 \| 17821 \| \| Trans. \| 1848 \| 2745 \| |

Note. Obs. = observed classes, Pred. = predicted classes. Cont. = Continued. Trans. = Transferred

1. Institutional + behavioral engagement data

| Uni | Elastic Net | Random Forest |
| --- | --- | --- |
| 1 | \|  \|  \| Pred. \| \| \| --- \| --- \| --- \| --- \| \|  \|  \| Cont. \| Trans. \| \| Obs. \| Cont. \| 288 \| 71 \| \| Trans. \| 45 \| 72 \| | \|  \|  \| Pred. \| \| \| --- \| --- \| --- \| --- \| \|  \|  \| Cont. \| Trans. \| \| Obs. \| Cont. \| 324 \| 35 \| \| Trans. \| 41 \| 76 \| |
| 2 | \|  \|  \| Pred. \| \| \| --- \| --- \| --- \| --- \| \|  \|  \| Cont. \| Trans. \| \| Obs. \| Cont. \| 1384 \| 309 \| \| Trans. \| 169 \| 148 \| | \|  \|  \| Pred. \| \| \| --- \| --- \| --- \| --- \| \|  \|  \| Cont. \| Trans. \| \| Obs. \| Cont. \| 1524 \| 169 \| \| Trans. \| 195 \| 122 \| |
| 3 | \|  \|  \| Pred. \| \| \| --- \| --- \| --- \| --- \| \|  \|  \| Cont. \| Trans. \| \| Obs. \| Cont. \| 1881 \| 263 \| \| Trans. \| 198 \| 205 \| | \|  \|  \| Pred. \| \| \| --- \| --- \| --- \| --- \| \|  \|  \| Cont. \| Trans. \| \| Obs. \| Cont. \| 2028 \| 116 \| \| Trans. \| 248 \| 155 \| |
| 4 | \|  \|  \| Pred. \| \| \| --- \| --- \| --- \| --- \| \|  \|  \| Cont. \| Trans. \| \| Obs. \| Cont. \| 19716 \| 20753 \| \| Trans. \| 1280 \| 3313 \| | \|  \|  \| Pred. \| \| \| --- \| --- \| --- \| --- \| \|  \|  \| Cont. \| Trans. \| \| Obs. \| Cont. \| 32072 \| 8397 \| \| Trans. \| 1876 \| 2717 \| |

Note. Obs. = observed classes, Pred. = predicted classes. Cont. = Continued. Trans. = Transferred

*Figure S1.* F1, TNR, and TPR performance for cross-university predictions (RQ3)

F1:


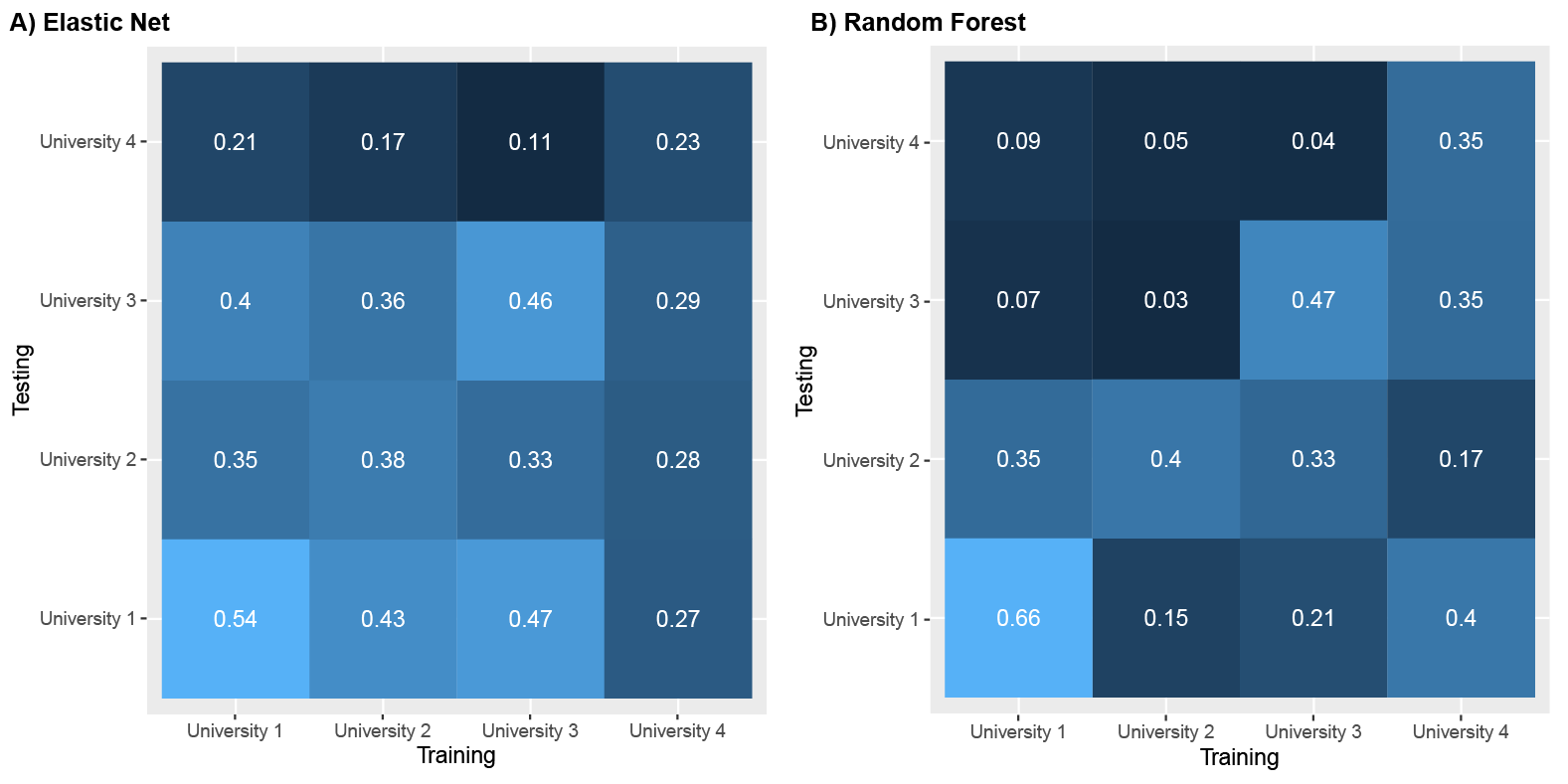


True Negative Rate (TNR):


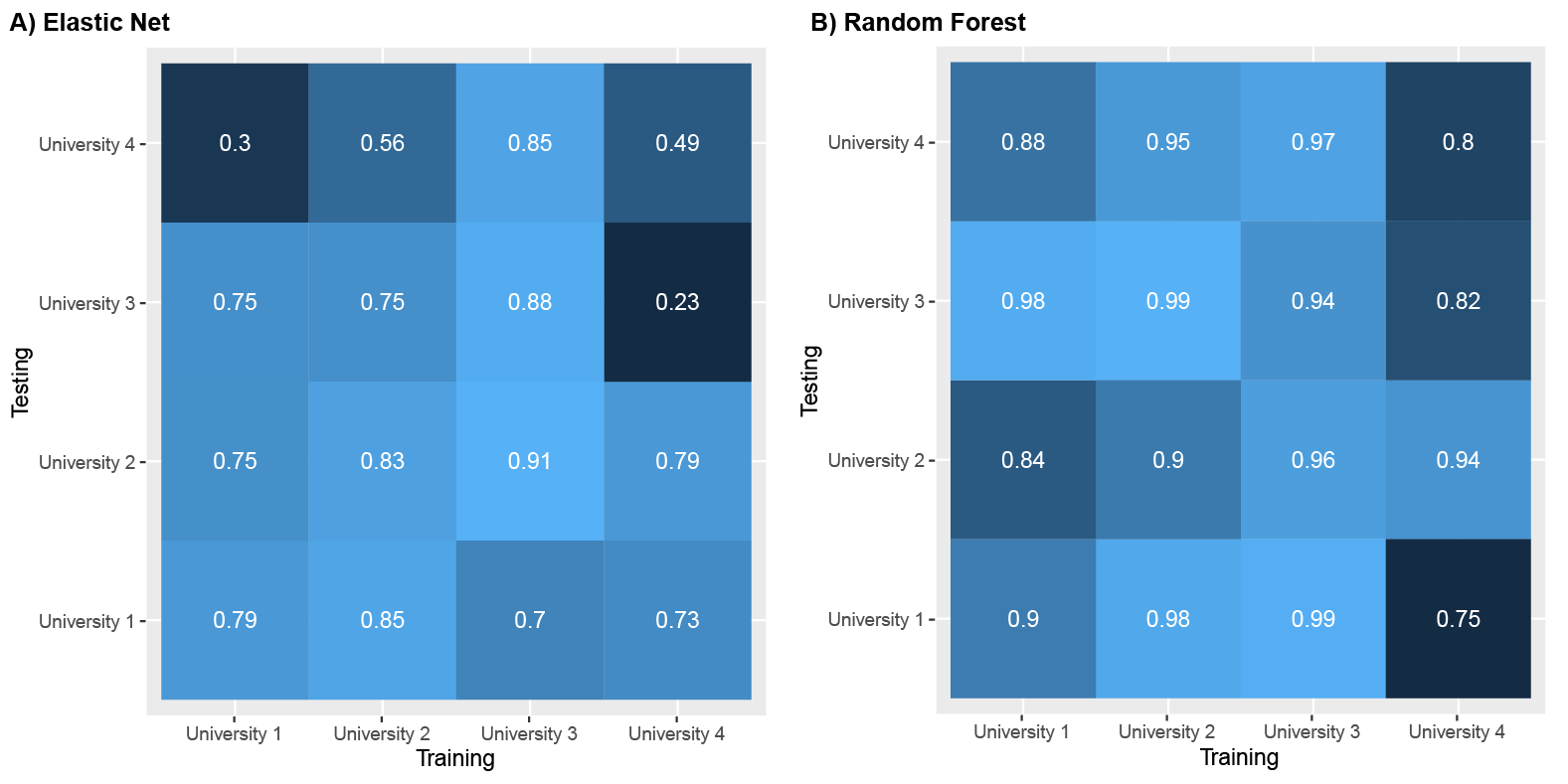


True Positive Rate (TPR):


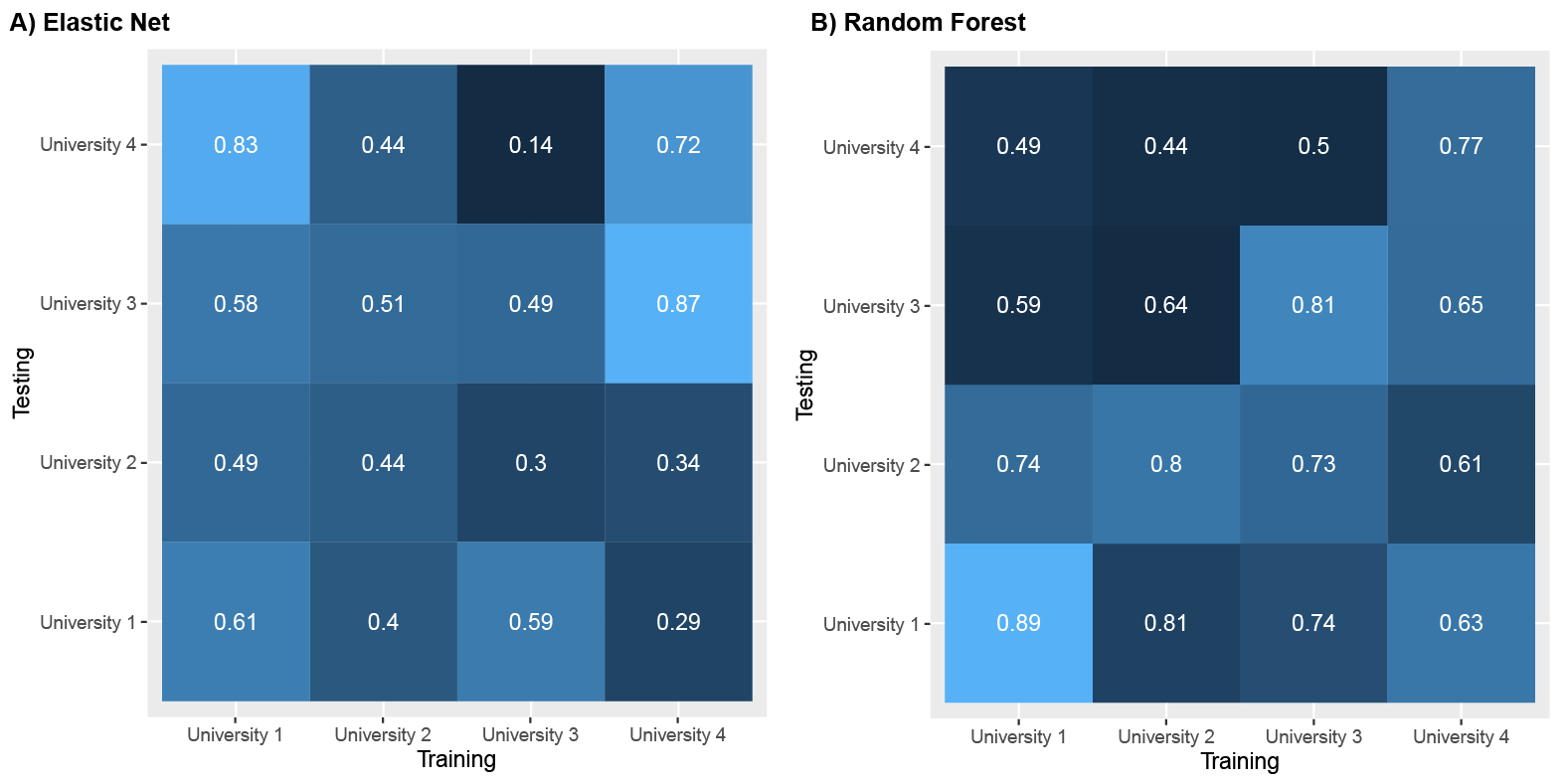

Supplement: Supplementary file 1 — Supplementary Information. [file 41598_2023_32484_MOESM1_ESM.docx]
